# CA19‑9 is a significant prognostic factor in stage III gastric cancer patients undergoing radical gastrectomy

**DOI:** 10.1186/s12893-024-02324-3

**Published:** 2024-01-23

**Authors:** Xiao Ma, Xiaohua Zhou, Jiaxuan Guo, Xinyu Feng, Mengmeng Zhao, Peng Zhang, Chong Zhang, Shuai Gong, Nai Wu, Yi Zhang, Xiuzhong Zhang, Zeqiang Ren, Pengbo Zhang

**Affiliations:** 1 Department of General Surgery, Affiliated Hospital of Xuzhou Medical University, #99 Huaihai Xi Road, JiangSu, Xuzhou 221002 China; 2 Department of General Surgery, Nanjing Gaochun People’s Hospital, #53 Maoshan Road, Gaochun Economic Development Zone, JiangSu, Nanjing 211300 China

**Keywords:** Gastric cancer, CA19-9, Prognosis, Adjuvant chemotherapy

## Abstract

**Background:**

Due to the great heterogeneity of gastric cancer (GC), the prognosis of patients within a stage is very different. Therefore, it is necessary to identify the high risk factors for postoperative recurrence and metastasis and take appropriate therapeutic strategies to improve the prognosis of patients. In this study, we aimed to explore the prognostic significance of preoperative and postoperative serum carcinoembryonic antigen (CEA), carbohydrate antigen 19 − 9 (CA19-9) and carbohydrate antigen 72 − 4 (CA72-4) in patients with stage I, II and III GC who underwent radical gastrectomy.

**Methods:**

A total of 580 patients who underwent curative surgical resection and had not received neoadjuvant chemotherapy were included in this study. The relationship between clinicopathological features and recurrence was analysed. Survival analysis was performed by Kaplan–Meier curve. Univariate and multivariate Cox regression analyses were performed to determine prognostic factors in GC patients.

**Results:**

Among patients with stage III GC, the recurrence free survival (RFS) and overall survival (OS) of patients with CA19-9>35 U/mL were significantly lower than those with CA19-9 ≤ 35 U/mL; CA19-9 was always a significant independent marker. CEA and CA72-4 were sometime useful to predict RFS or OS alternatively in the pre- or postoperative period. The only other independent significant factors for prognosis in our study were lymph node metastases for RFS and postoperative adjuvant chemotherapy for OS.

**Conclusion:**

Preoperative and postoperative CA19-9 values are independent risk factors for predicting prognosis in stage III GC after curative gastrectomy.

**Supplementary Information:**

The online version contains supplementary material available at 10.1186/s12893-024-02324-3.

## Introduction

Gastric cancer (GC) is a heterogeneous and highly aggressive malignant tumor, and it is the fourth leading cause of cancer-related death worldwide [1]. Because of its insidious onset, most GC patients are already in the advanced stage at the time of diagnosis rendening treatment difficult, with a high rate of recurrence and relatively poor prognosis [2, 3]. Radical gastrectomy with adjuvant chemotherapy is presently considered a standard treatment for stage II/III advanced GC patients [4]. However, the prognosis of GC patients undergoing radical gastrectomy remains poor [5–7]. Recurrence and distant metastasis are the main reason for death of GC patients after curative resection. Due to prognostic heterogeneity within each stage, identification of specific risk factors and accurate prediction for recurrence is warranted; this would help in performing appropriate intensive adjuvant therapies and in surveillance planning [8].

Serum tumor markers reflect tumor characteristics and burden generated by the tumor itself or in response to tumor cells [9, 10]. They have been used for GC screening and surveillance of relapse after radical surgery [11]. An increase in tumor markers is usually detected before clinical recurrence. Although many studies have reported that preoperative carcinoembryonic antigen (CEA), carbohydrate antigen 19 − 9 (CA19-9) and carbohydrate antigen 72 − 4 (CA72-4) can be used as prognostic factors [12, 13], their association with the prognosis and recurrence in different GC stages remains unclear. Thus, the aim of this study was to explore the prognostic significance of serum CEA, CA19-9 and CA72-4 levels for GC patients with stage I, II and III who underwent radical gastrectomy and their relationship with recurrence.

## Materials and methods

### Study population

A total of 580 GC patients who underwent radical gastrectomy at the Affiliated Hospital of XuZhou Medical University and Nanjing Gaochun People^,^s Hospital from August 2018 to August 2021 were retrospectively enrolled in this study. A diagnosis of GC was confirmed by histopathology, and TNM stage was staged in accordance with the 8th Edition of the American Joint Committee on Cancer (AJCC) classification. The protocol was approved by the Hospital Ethics Committee, and written informed consent was signed by all patients. Exclusion criteria included: (1) patients with previous or concomitant other cancer; (2) patients with non-radical surgery or distant metastases; (3) patients with prior history of neoadjuvant therapy; and (4) patients without key clinical variables or follow-up data.

### Data collection and follow-up

Clinicopathological data including sex, age, tumor location, tumor size, pathologic type, degree of differentiation, nerve and vascular invasion, lymph node status and postoperative chemotherapy were recorded. Preoperative serum CEA, CA19-9 and CA72-4 levels (< 1 weeks before surgery) were recorded. The CA19-9 value less than 1 U/mL was excluded. For patients with recurrence, postoperative tumor marker levels (> 3 months after surgery) before recurrence were recorded during follow-up. For patients without recurrence, the postoperative tumor marker levels (> 3 months after surgery) before the end of follow-up were recorded during follow-up. The normal reference values of CEA, CA19-9 and CA72-4 were 5.0ng/ml, 35 U/ml and 6.9 U/ml, respectively. A test value above normal was considered positive. The positive results were defined when CEA (+) > 5.0 ng/mL, CA19-9 (+) > 35 U/mL and CA72-4 (+) > 6.9 U/mL. During the follow-up period, recurrence was confirmed by postoperative serum tumor biomarker levels, enhanced CT, gastroscopy and other relevant examinations. Overall survival (OS) was defined as the time from the date of surgery to the date of death or the end of follow-up (August 2022), and recurrence free survival (RFS) was the time interval between the date of surgery and the date of objective tumor relapse or the end of follow-up.

### Statistical analysis

Categorical variables were analysed using Pearson chi-square tests or Fisher’s exact test. Kaplan-Meier survival curves were obtained by GraphPad Prism 8 software and compared with the log rank test. Univariate and multivariate analyses were performed to evaluate the independent risk factors associated with OS and PFS using the Cox proportional hazards model. The statistical analysis was performed using SPSS 26.0 (SPSS, Inc). The *P* value less than 0.05 was considered to be statistically significant.

## Results

### Patient characteristics

A total of 580 patients underwent radical gastrectomy, including 115 patients in stage I, 163 patients in stage II, and 302 patients in stage III. Of these patients, 423 were male and 157 were female. The median age was 65 years (range 32–86 years). The median follow-up duration was 29 months (range 9–45 months), and 86 patients (14.8%) died and 97 cases (16.7%) had a tumor recurrence.

Totally 9 patients in stage I, 73 patients in stage II, and 140 patients in stage III received postoperative adjuvant chemotherapy (PAC), of whom 1, 11 and 85 patients relapsed in stage I, II and III, respectively. The PAC regimen was SOX regimen (S-1 combined with oxaliplatin).

### Correlation between CEA, CA19-9 and CA72-4 levels and recurrence in GC patients with stage I, II and III

**Fig. 1:**
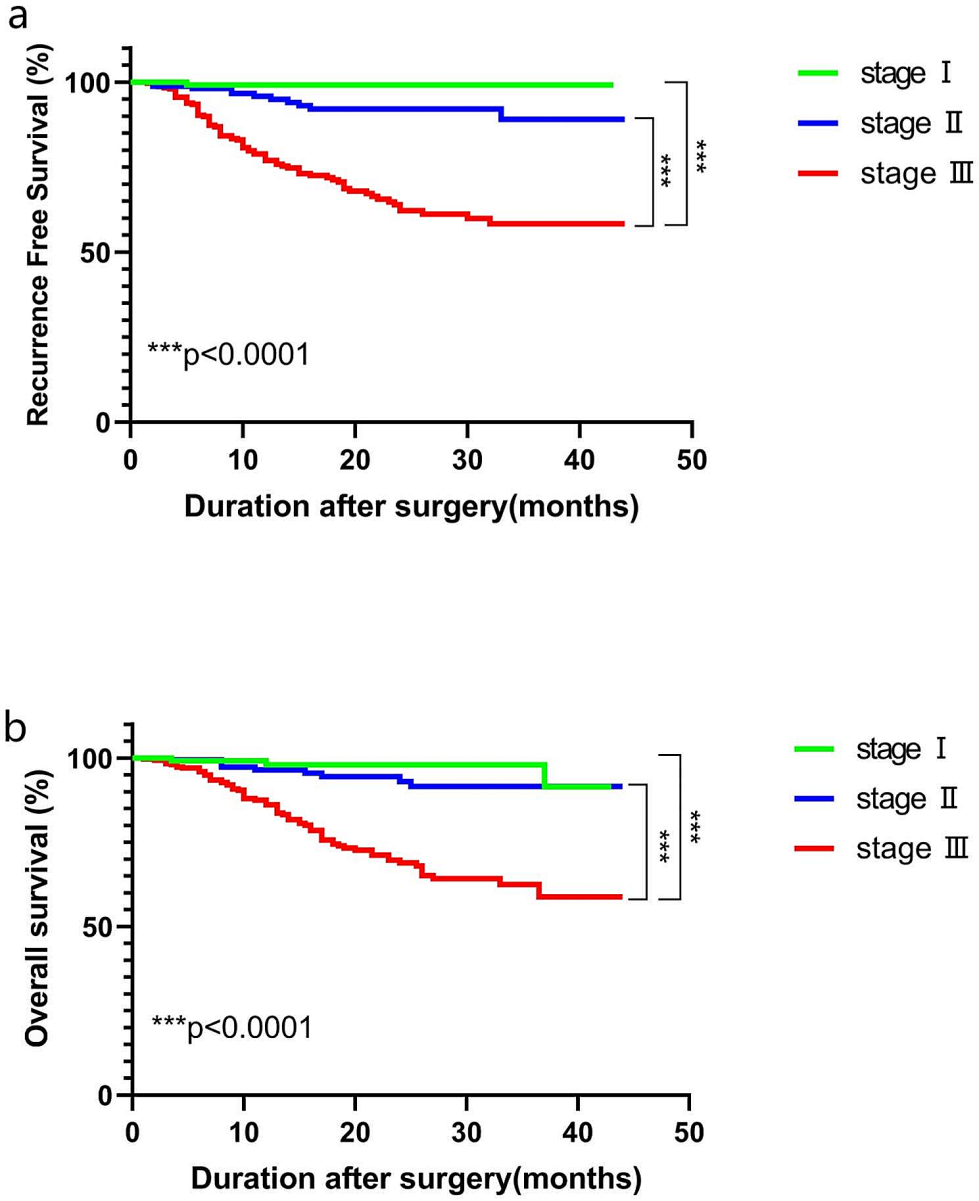
RFS curves (**a**) and OS curves (**b**) of GC patients with stage I, II and III

The proportions of patients with preoperative and postoperative CEA (+) and CA19-9 (+) levels were significantly higher in those at stage III and with recurrence than that in patients at stage I and II (Tables 1 and 2). In addition, The RFS values were 99.1%, 89.1% and 58.3% for patients with stage I, II and III GC, respectively (Fig. 1a). Moreover, the OS values were 98.0%, 91.5% and 62.4% for GC patients with stage I, II and III, respectively (Fig. 1b). Kaplan-Meier survival analysis revealed that GC patients with stage III had a worse prognosis than those with stage I and II (Fig. 1). Further analysis showed that stage III GC patients with high pre- or postoperative levels of CEA, CA19-9 and CA72-4 trended to have lower RFS and OS, however, this difference was not significant in stage I and II GC patients (Fig. S). Therefore, we further analyzed the role of CEA, CA19-9 and CA72-4 levels in postoperative recurrence of patients with stage III GC.

**Table 1:** Positive rates of serum CEA, CA19-9 and CA72-4 levels

groups	Case		preoperative				postoperative	
		CEA(+)	CA19-9(+)	CA72-4(+)		CEA(+)	CA19-9(+)	CA72-4(+)
stage I and II GC (%)^a^	278	48(17.3)	27(9.7)	45(16.2)		37(13.3)	10(3.6)	23(8.3)
stage III (%)^a^	302	90(29.8)*	67(22.2)*	60(19.9)		74(24.5)*	42 (13.9)*	38(12.6)

GC: Gastric Cancer; CEA(+): CEA > 5 ng/mL; CA19-9(+): CA19-9 > 35 U/mL; CA72-4(+): CA72-4 > 6.9 U/mL; ^a^Pearson chi-squared test or Fisher’s exact test; compared with the stage I and II GC group, *P < 0.05

**Table 2:** Recurrence rates in patients with positive tumor markers

tumor markers	stage I and II GC	patients with recurrence	stage III GC	patients with recurrence
preoperative(%)^a^				
CEA(+)	48	2(4.2)	90	35(38.9)*
CA19-9(+)	27	2(7.4)	67	32(47.8)*
CA72-4(+)	45	4(8.9)	60	21(35.0)*
postoperative(%)^a^				
CEA(+)	37	1(2.7)	74	36(48.6)*
CA19-9(+)	10	2(20.0)	42	28(66.7)*
CA72-4(+)	23	2(8.7)	38	24(63.2)*

GC: Gastric Cancer; CEA(+): CEA > 5 ng/mL; CA19-9(+): CA19-9 > 35 U/mL; CA72-4(+): CA72-4 > 6.9 U/mL; ^a^Pearson chi-squared test or Fisher’s exact test; compared with the stage I and II GC group, *P < 0.05

The association between cancer recurrence and clinicopathological features in patients with stage III GC was summarized in Table 3. Preoperative CEA and CA19-9 levels, postoperative CEA, CA19-9 and CA72-4 levels, lymph node metastasis, and differentiation were associated with cancer recurrence. However, there was no significant association between recurrence and preoperative CA72-4 levels. The recurrence rates of patients with CEA > 5 ng/mL or CA19-9 > 35 U/mL were significantly higher than that of patients with CEA ≤ 5ng/mL or CA19-9 ≤ 35 U/mL(preoperative, 38.9% vs. 23.6%, 47.8% vs. 22.6%, respectively; postoperative, 48.6% vs. 21.5%, 66.7% vs. 21.9%, respectively; *P* < 0.05, Table 3).

**Table 3:** The relationship of recurrence and clinicopathological parameters in stage III GC

Clinicopathological parameters	Cases	Patients with recurrence (*n* = 85)	Patients without recurrence (*n* = 217)	χ^2^	*p*
Preoperative CEA(ng/mL)				7.317	**0.007**
≤ 5	212	50(23.6%)	162(76.4%)		
> 5	90	35(38.9%)	55(61.1%)		
Preoperative CA19-9(U/mL)				16.381	**< 0.001**
≤ 35	235	53(22.6%)	182(77.4%)		
> 35	67	32(47.8%)	35(52.2%)		
Preoperative CA72-4(U/mL)				1.739	0.187
≤ 6.9	242	64(26.4%)	178(73.6%)		
> 6.9	60	21(35.0%)	39(65.0%)		
Age(years)				1.715	0.190
< 60	80	18(22.5%)	62(77.5%)		
≥ 60	222	67(30.2%)	155(69.8%)		
Sex				2.136	0.144
Female	82	18(22.0%)	64(78.0%)		
Male	220	67(30.5%)	153(69.5%)		
Tumor location				6.462	0.091
Upper	81	21(25.9%)	60(74.1%)		
Middle	54	21(38.9%)	33(61.1%)		
Lower	147	41(27.9%)	106(72.1%)		
Other	20	2*(20.0%)	18(80.0%)		
Tumor size (cm)				1.326	0.249
< 5	151	38(25.2%)	113(74.8%)		
≥ 5	151	47(31.1%)	104(68.9%)		
Depth of invasion				0.254	0.614
T2-T3	202	55(27.2%)	147(72.8%)		
T4	100	30(30.0%)	70(70.0%)		
Lymph node metastasis				6.697	**0.010**
N0-N1	52	7(13.5%)	45(86.5%)		
N2-N3	250	78(31.2%)	172(68.8%)		
Vascular invasion				3.537	0.060
Yes	234	72(30.8%)	162(69.2%)		
No	68	13(19.1%)	55(80.9%)		
Nerve invasion				0.241	0.624
Yes	247	71(28.7%)	176(71.3%)		
No	55	14(25.5%)	41(74.5%)		
Degree of differentiation				3.969	**0.046**
Moderately	31	4*(12.9%)	27(87.1%)		
Poorly	271	81(29.9%)	190(70.1%)		
Postoperative adjuvant chemotherapy				2.062	0.151
Yes	140	45(32.1%)	95(67.9%)		
No	162	40(24.7%)	122(75.3%)		
Postoperative CEA(ng/mL)				20.374	**< 0.001**
≤ 5	228	49(21.5%)	179(78.5%)		
> 5	74	36(48.6%)	38(51.4%)		
Postoperative CA19-9(U/mL)				35.794	**< 0.001**
≤ 35	260	57(21.9%)	203(78.1%)		
> 35	42	28(66.7%)	14(33.3%)		
Postoperative CA72-4(U/mL)				26.349	< 0.001
≤ 6.9	264	61(23.1%)	203(76.9%)		
> 6.9	38	24(63.2%)	14(36.8%)		

*Indicates that the theoretical frequency of the four-cell table is less than 5, and the statistical analysis was performed using Fisher exact probability method

### Univariate and multivariate analysis in patients with stage III GC

**Table 4:** Univariate and multivariate analyses of clinicopathological factors for RFS in stage III GC

Variables	β	Univariate		*P*-Value	β	Multivariate		*P*-Value
		Wald	HR(95%CI)			Wald	HR(95%CI)	
Preoperative CEA(ng/mL)								
≤ 5								
> 5	0.528	5.738	1.696(1.101–2.614)	0.017	0.37	2.473	1.448(0.913–2.296)	0.116
Preoperative CA19-9(U/mL)								
≤ 35								
> 35	1.049	21.688	2.853(1.835–4.436)	**< 0.001**	0.619	6.154	1.858(1.139–3.031)	**0.013**
Preoperative CA72-4(U/mL)								
≤ 6.9								
> 6.9	0.232	0.848	1.261(0.770–2.064)	0.848				
Age(years)								
≤ 60								
> 60	0.241	0.823	1.273(0.756–2.145)	0.364				
Gender								
Female								
Male	0.274	1.067	1.316(0.782–2.214)	0.302				
Tumor location		2.344		0.504				
Upper								
Middle	0.275	0.796	1.317(0.719–2.413)	0.372				
Lower	0.011	0.002	1.011(0.597–1.711)	0.967				
Other	-0.716	0.934	0.489(0.114–2.088)	0.334				
Tumor size(cm)								
<5								
≥ 5	0.376	2.955	1.456(0.949–2.236)	0.086				
Vascular invasion								
No								
Yes	0.401	1.766	1.493(0.827–2.695)	0.184				
Nerve invasion								
No								
Yes	0.191	0.428	1.211(0.682–2.148)	0.513				
Degree of differentiation								
Moderately								
Poorly	0.866	2.854	2.377(0.871–6.488)	0.091				
Depth of invasion								
T2-T3								
T4	0.076	0.111	1.078(0.691–1.683)	0.74				
Lymph node metastasis								
N0-N1								
N2-N3	1.009	6.523	2.743(1.265–5.951)	**0.011**	0.917	5.205	2.502(1.138–5.503)	**0.023**
Postoperative adjuvant chemotherapy(PAC)								
No								
Yes	0.123	0.322	1.131(0.739–1.732)	0.571				
Postoperative CEA(ng/mL)								
≤ 5								
> 5	0.791	12.946	2.205(1.433–3.392)	**< 0.001**	0.029	0.011	1.030(0.593–1.787)	0.917
Postoperative CA19-9(U/mL)								
≤ 35								
> 35	1.475	40.016	4.370(2.767–6.901)	**< 0.001**	0.902	9.598	2.464(1.393–4.359)	**0.002**
Postoperative CA72-4(U/mL)								
≤ 6.9								
> 6.9	1.252	26.774	3.497(2.2176–5.619)	**< 0.001**	0.938	11.146	2.555(1.473–4.432)	**0.001**

The univariate analysis for RFS showed that preoperative CEA and CA19-9 levels, lymph node metastasis, postoperative CEA, CA19-9 and CA72-4 levels were significantly correlated with RFS in stage III GC (Table 4, all *p* < 0.05). Moreover, the univariate analysis for OS showed that preoperative CA19-9 levels, tumor size, nerve invasion, PAC, and postoperative CA19-9 and CA72-4 levels were significantly associated with OS in stage III GC (Table 5, all *p* < 0.05). Furthermore, in multivariate analysis, preoperative CA19-9 levels (HR:1.858; 95% CI:1.139–3.031; *p* = 0.013), lymph node metastasis (HR:2.502; 95% CI:1.138–5.503; *p* = 0.023), and postoperative CA19-9 (HR:2.464; 95% CI:1.393–4.359; *p* = 0.002) and CA72-4 (HR:2.555; 95% CI:1.473–4.432; *p* = 0.001) levels were significant independent prognosis factors for RFS in stage III GC; Preoperative CA19-9 levels (HR:1.878; 95% CI:1.121–3.147; *p* = 0.017), PAC (HR:0.550; 95% CI: 0.342–0.886; *p* = 0.014), and postoperative CA19-9 levels (HR:1.881; 95% CI: 1.081–3.270; *p* = 0.025) were significant independent factors for OS in stage III GC. Thus, the results indicated that CEA and CA72-4 were sometimes significant in preoperative or postoperative for RFS or OS. CA19-9 was always a significant factor both in univariate and multivariate analysis and for RFS or OS.

**Table 5:** Univariate and multivariate analyses of clinicopathological factors for OS in stage III GC

Variables	β	Univariate		*P*-Value	β	Multivariate		*P*-Value
		Wald	HR(95%CI)			Wald	HR(95%CI)	
Preoperative CEA(ng/mL)								
≤ 5								
> 5	0.175	0.517	1.191(0.739–1.920)	0.472				
Preoperative CA19-9(U/mL)								
≤ 35								
> 35	0.897	13.65	2.451(1.523–3.945)	**< 0.001**	0.63	5.726	1.878(1.121–3.147)	**0.017**
Preoperative CA72-4(U/mL)								
≤ 6.9								
> 6.9	0.169	0.387	1.184(0.696–2.014)	0.534				
Age(years)								
≤ 60								
> 60	0.509	2.592	1.663(0.895–3.088)	0.107				
Gender								
Female								
Male	-0.234	0.845	0.791(0.481–1.303)	0.358				
Tumor location		1.962		0.58				
Upper								
Middle	-0.268	0.651	0.765(0.399–1.467)	0.42				
Lower	-0.374	1.871	0.688(0.402–1.176)	0.171				
Other	-0.099	0.033	0.906(0.312–2.629)	0.855				
Tumor size(cm)								
<5								
≥ 5	0.593	6.167	1.810(1.133–2.890)	**0.013**	0.324	1.69	1.382(0.848–2.252)	0.194
Vascular invasion								
No								
Yes	0.175	0.328	1.191(0.654–2.169)	0.567				
Nerve invasion								
No								
Yes	0.785	3.908	2.193(1.007–4.779)	**0.048**	0.756	3.558	2.129(0.971–4.669)	0.059
Degree of differentiation								
Moderately								
Poorly	0.42	0.822	1.522(0.614–3.773)	0.365				
Depth of invasion								
T2-T3								
T4	0.171	0.506	1.186(0.741-1.900)	0.477				
Lymph node metastasis								
N0-N1								
N2-N3	0.726	3.73	2.067(0.989–4.318)	0.053				
Postoperative adjuvant chemotherapy(PAC)								
No								
Yes	-0.515	4.661	0.598(0.374–0.954)	**0.031**	-0.597	6.043	0.550(0.342–0.886)	**0.014**
Postoperative CEA(ng/mL)								
≤ 5								
> 5	0.267	1.181	1.307(0.807–2.117)	0.277				
Postoperative CA19-9(U/mL)								
≤ 35								
> 35	1.104	18.366	3.015(1.820–4.995)	**< 0.001**	0.632	5.006	1.881(1.081–3.270)	**0.025**
Postoperative CA72-4(U/mL)								
≤ 6.9								
> 6.9	0.828	9.295	2.288(1.344–3.897)	**0.002**	0.555	3.611	1.741(0.983–3.085)	0.057

### Prognostic significance of preoperative and postoperative CA19-9 levels in patients with stage III GC

**Fig. 2:**
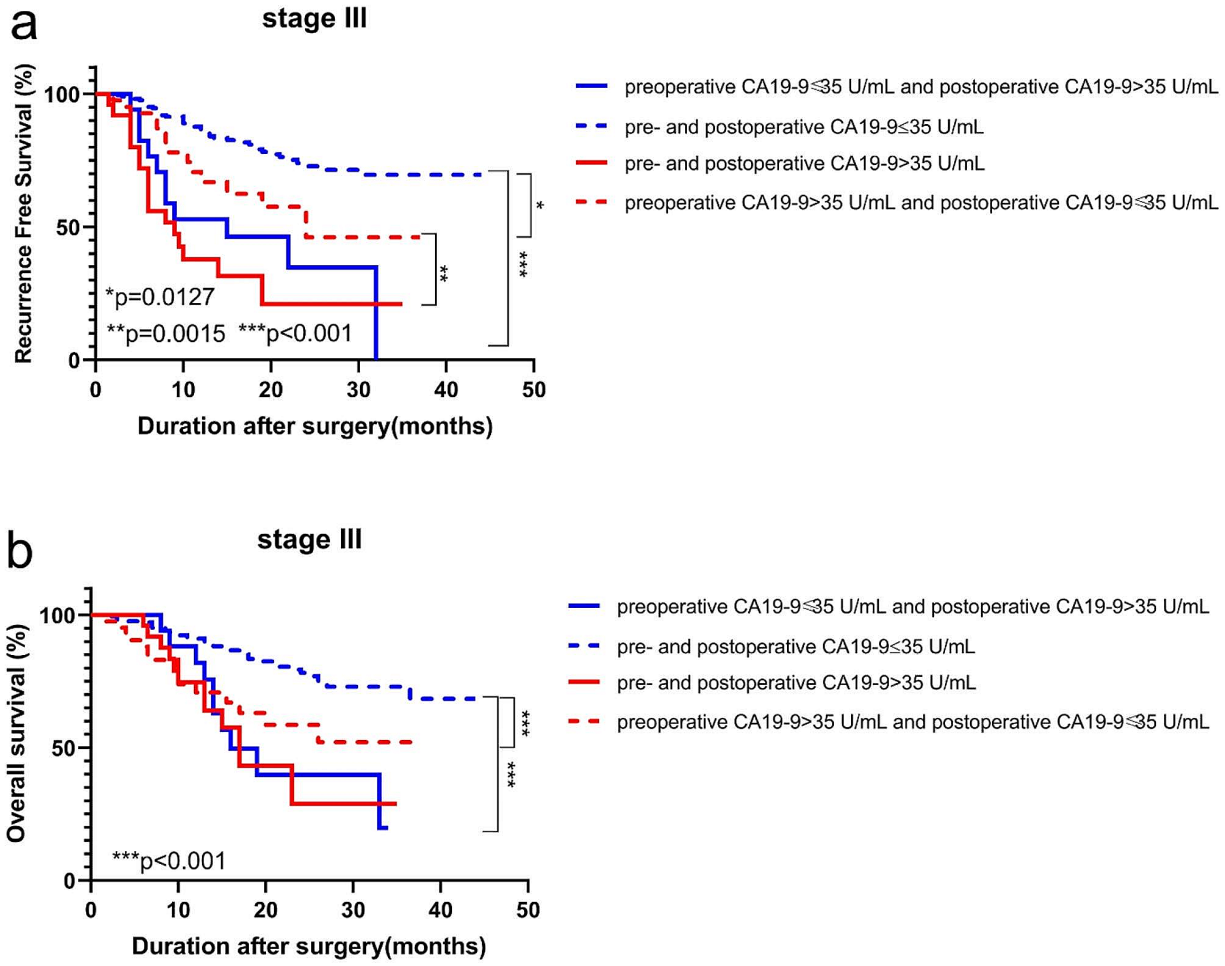
Prognostic impact of pre- and postoperative CA19-9 levels in stage III GC. RFS curves (**a**) and OS curves (**b**)

Multivariate analysis had showed that preoperative and postoperative CA19-9 levels were the significantly independent factors for RFS and OS. We further analyzed the prognostic impact of preoperative and postoperative CA19-9 levels in stage III GC. The patients were categorized into the following four groups according to preoperative and postoperative CA19-9 levels: preoperative CA19-9 ≤ 35 U/mL and postoperative CA19-9 > 35 U/mL, pre- and postoperative CA19-9 > 35 U/mL, pre- and postoperative CA19-9 ≤ 35 U/mL, preoperative CA19-9 > 35 U/mL and postoperative CA19-9 ≤ 35 U/mL. The Kaplan-Meier curves for RFS and OS in connection with CA19-9 values were shown in Fig. 2. The RFS of patients with preoperative high CA 19 - 9 that become negative after surgery were longer than that of patients always above 35 U/mL (Fig. 2a, *p* = 0.0015). The RFS of patients always under 35 U/mL were also longer than preoperative low CA19-9 that become positive after surgery (Fig. 2a, *p* < 0.001). The same trend was observed for OS (Fig. 2b). Thus, the results indicated that the patients with a elevated postoperative CA19-9 level had a poor prognosis.

**Fig. 3:**
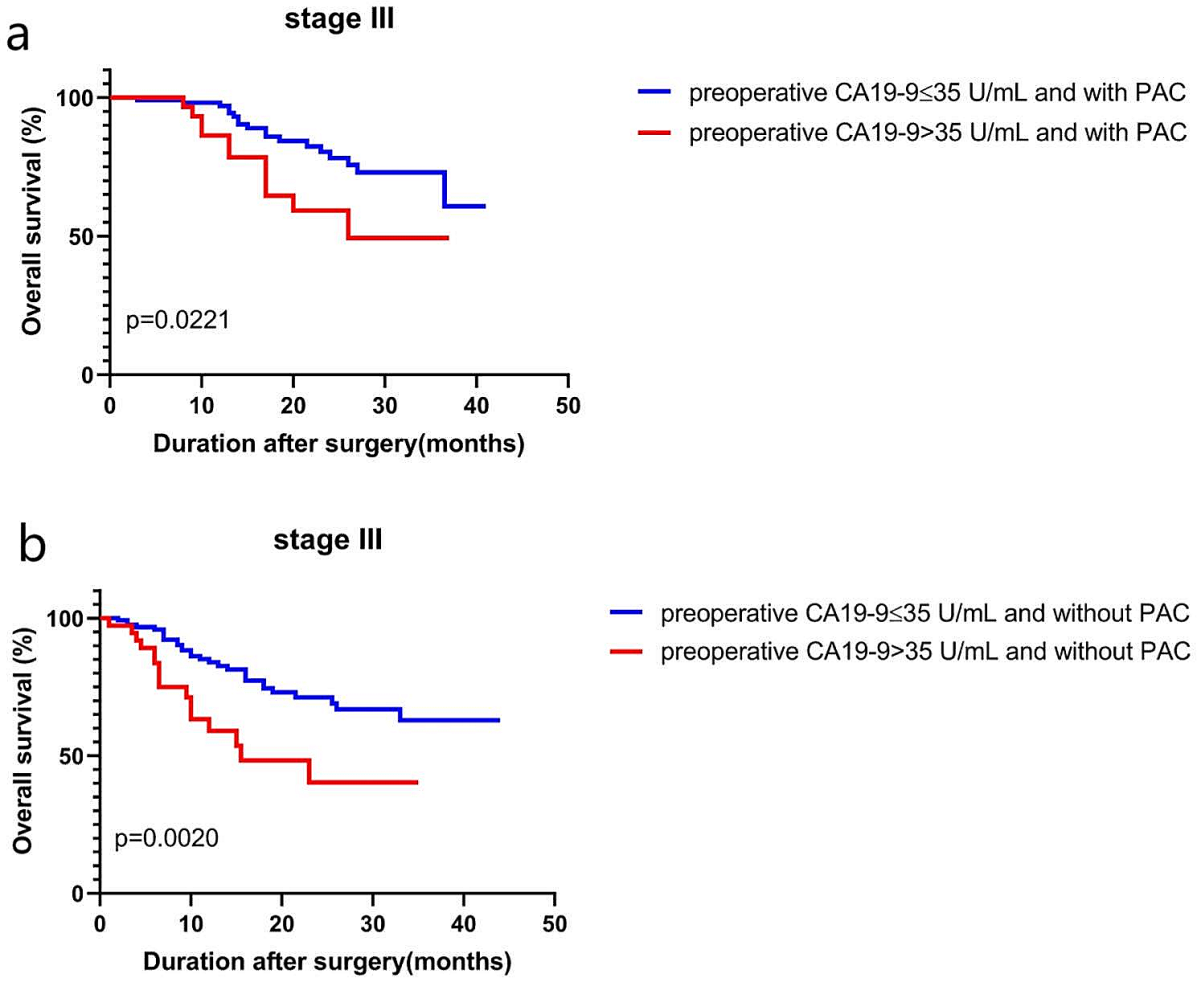
Prognostic impact of preoperative CA19-9 levels of stage III GC patients with (**a**) or without (**b**) postoperative adjuvant chemotherapy (PAC)

The RFS and OS of patients with preoperative high CA19-9 that become negative after surgery were lower than patients always under 35 U/mL (Fig. 2a-b, *p* = 0.0127, *p* < 0.001, respectively). Excluding the factor of postoperative adjuvant chemotherapy (PAC), the OS of patients with preoperative high CA19-9 was also significantly lower than that of patients with preoperative low CA19-9 (Fig. 3a-b, *p* = 0.0021, *p* = 0.0020, respectively). Thus, preoperative high CA19-9 levels also indicated poor outcomes.

## Discussion

Gastric cancer (GC) is a highly heterogeneous and aggressive malignant tumor with poor prognosis. Due to prognostic heterogeneity within each stage, identification of high-risk subgroups and individualized treatment is warranted. Previous study showed that tumor markers can be used in the diagnosis, prognosis, recurrence prediction and treatment response of GC [14]. They can also reflect tumor progression and burden. Therefore, this study investigated the prognostic value of serum CEA, CA19-9 and CA72-4 levels and their relationship with recurrence in patients with stage I, II and III GC who underwent radical gastrectomy.

Advanced gastric cancer patients are more likely to relapse and has a worse outcome after radical gastrectomy. Tumor markers have been reported as valuable predictors for the prognosis of gastric cancer. Our study suggested that CEA was significant only in univariate for RFS and CA72-4 was significant in univariate and multivariate for RFS at stage III GC. The only marker always significant for RFS and OS was CA19-9 that can be a useful marker for predicting outcome compared with CEA and CA72-4.

Carbohydrate antigen 19 − 9 (CA19-9), also called sialyl Lewis antigen A, has been widely used as a tumor-associated biomarker for the treatment of gastrointestinal malignancies, especially pancreatic cancer. Due to Lewis gene dysfunction or fucosyltransferase deficiency, approximately 5–10% of individuals are Lewis antigen negative, with no or low secretion of CA19-9 [15–17]. Therefore, we excluded GC patients with a CA19-9 value of less than 1 U/mL as in previous studies. Most previous studies only focused on the prognostic value of preoperative CA19-9 in GC. Few studies reported the value of pre- and postoperative CA19-9 in predicting prognosis and recurrence in stage III GC. A meta-analysis of 5,072 GC patients also showed that elevated serum CA19-9 was associated with poorer OS [18] as reported by many other studies [19–22] especially in stage III GC [23] or in association with pT and pN stage [24]. We did not find preoperative CEA or CA72-4 levels, pT stage were independent prognostic factors for OS and RFS. However, we found that pre- and postoperative CA19-9 were significant independent prognosis factors for RFS and OS in stage III GC.

In munivariate analysis, lymph node metastasis, postoperative CA72-4 were also significant independent factors for RFS in stage III GC. Eom BW et al. reported that LN metastasis and venous invasion were independent predictors of early recurrence [25]. Kang WM et al. also reported that patient age, pT stage, pN stage, Lauren histotype, lymphovascular invasion, intraoperative chemotherapy, and postoperative chemotherapy were independent predictors of early recurrence in patients with pT2-4a stage GC [26]. Wakatsuki Ket al. also reported that pN ≥ 14 and preoperative CA19-9 were independent risk factors for ERec (early recurrence within 12 months) after curative gastrectomy in pStage III GC [27]. A previous study of 1179 GC patients showed that pre- or postoperative CA72-4 was independently associated with shorter OS and RFS [28]. Consistent with previous findings, our study also suggested that lymph node metastasis was a risk factor for recurrence in GC patients. However, in our study preoperative CA72-4 was not an independent factors for RFS and OS in stage III GC, so further study is needed.

Multivariate analysis also showed that postoperative adjuvant chemotherapy (PAC) was a significant independent protective factor for OS in stage III GC. This indicated that PAC was beneficial to the prognosis of stage III GC patients. A previous study reported that adjuvant chemotherapy can improve the survival rate and disease-free survival rate of GC patients, and reduce the relapse rate after curative resection [29]. Randomized phase III trials showed that postoperative adjuvant therapy with S-1 or S-1 plus docetaxel could improve OS and RFS in patients with stage III GC who had undergone D2 gastrectomy [30, 31]. In addition, stage III GC patients with preoperative high CA19-9 that become negative after surgery had longer RFS and OS, but lower than patients always under 35 U/ml. Therefore, monitor the preoperative and postoperative CA19-9 levels in stage III GC patients is of great value for evaluating the treatment effect, predicting recurrence and prognosis. Due to preoperative or postoperative high CA19-9 levels all indicated poor outcomes. Thus, intensive postoperative anticancer management should be received.

This study had a few limitations. First, this was a retrospective study from two institutions with a possible selection bias and a relatively small samples. Second, follow-up period was short, and some cases were followed up for less than 3 years. Third, the comorbidities and postoperative complications were not investigated in this study, which could also affect the prognosis. Thus, a prospective, multicentre study with longer follow-up period is urgently needed for further investigation.

## Conclusions

This study showed preoperative and postoperative CA19-9 levels to be independent risk factors for predicting prognosis in stage III GC after curative gastrectomy. Patients with higher CA19-9 values should hence be considered for receiving intensive perioperative anticancer management following curative resections owing to the poor prognosis.

### Electronic supplementary material

Below is the link to the electronic supplementary material.

**Supplementary Material 1: Figure S** Prognostic impact of serum tumor markers. A and B: Overall and recurrence-free survival curves according to preoperative CEA, CA19-9 and CA72-4 levels; C and D: Overall and recurrence-free survival curves according to postoperative CEA, CA19-9 and CA72-4 levels. CEA: Carcinoembryonic antigen; CA19-9: Carbohydrate antigen 19-9. CA72-4: Carbohydrate antigen 72-4

## Data Availability

The datasets of the current study are available from the corresponding author upon reasonable request.
