# Supplementary material for: CA19‑9 is a significant prognostic factor in stage III gastric cancer patients undergoing radical gastrectomy
Source: BMC Surg. 2024 Jan 23;24:31. doi: 10.1186/s12893-024-02324-3 (PMC10804529; doi:10.1186/s12893-024-02324-3)
Supplement: Supplementary file 1 — Supplementary Material 1: Figure S Prognostic impact of serum tumor markers. A and B: Overall and recurrence-free survival curves according to preoperative CEA, CA19-9 and CA72-4 levels; C and D: Overall and recurrence-free survival curves according to postoperative CEA, CA19-9 and CA72-4 levels. CEA: Carcinoembryonic antigen; CA19-9: Carbohydrate antigen 19-9. CA72-4: Carbohydrate antigen 72-4 [file 12893_2024_2324_MOESM1_ESM.docx]

**A**  preoperative level (OS)


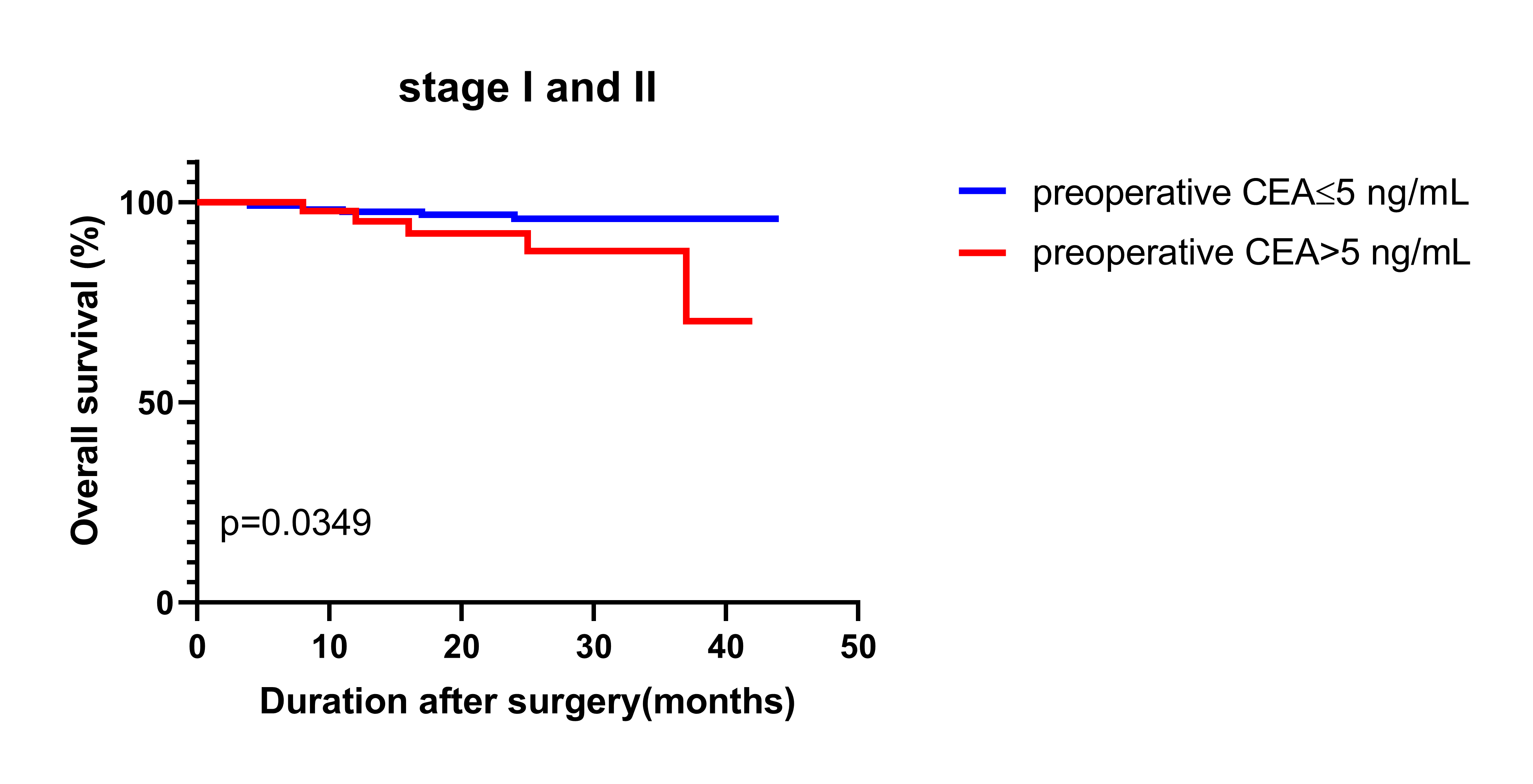


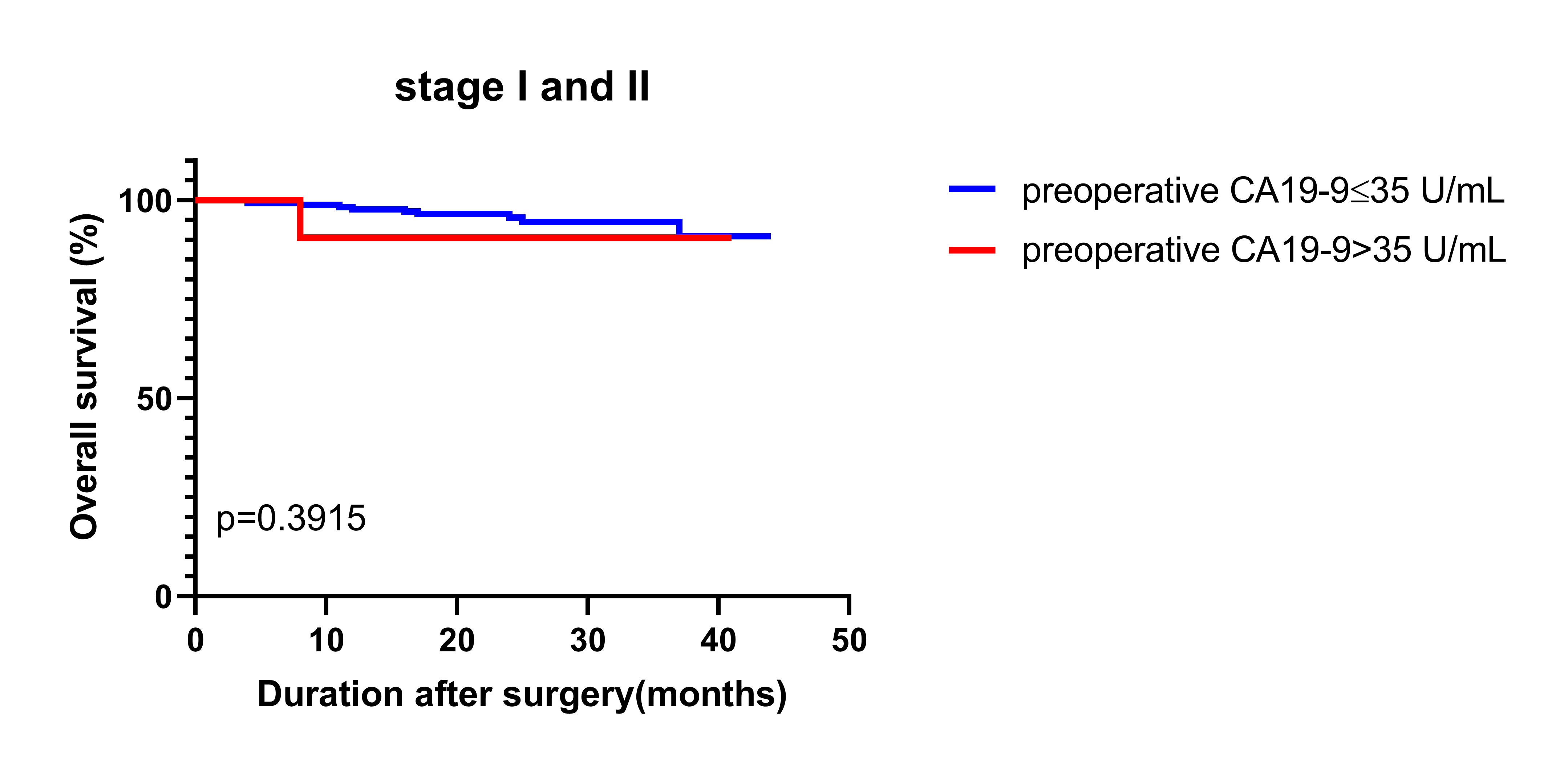


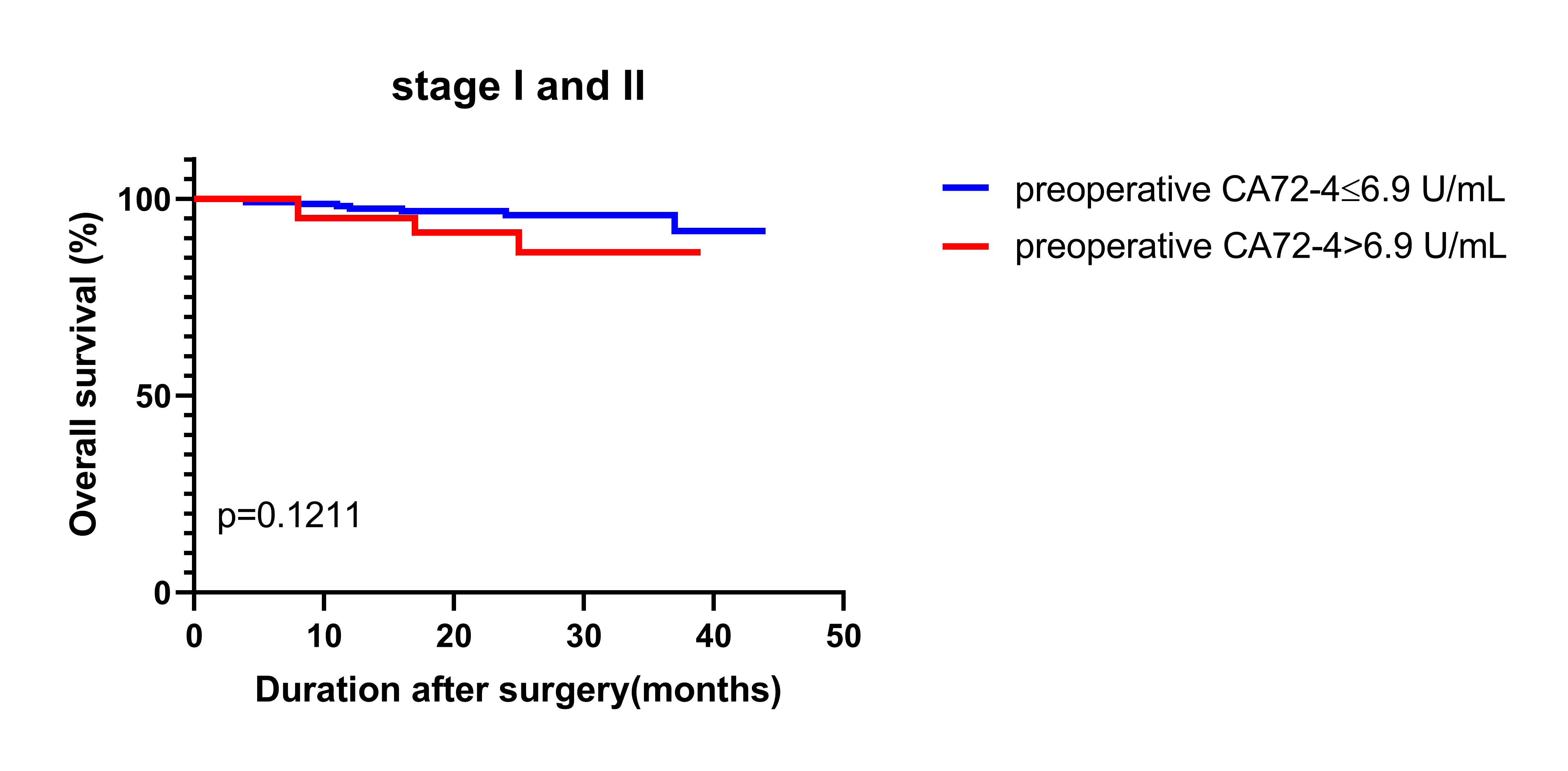


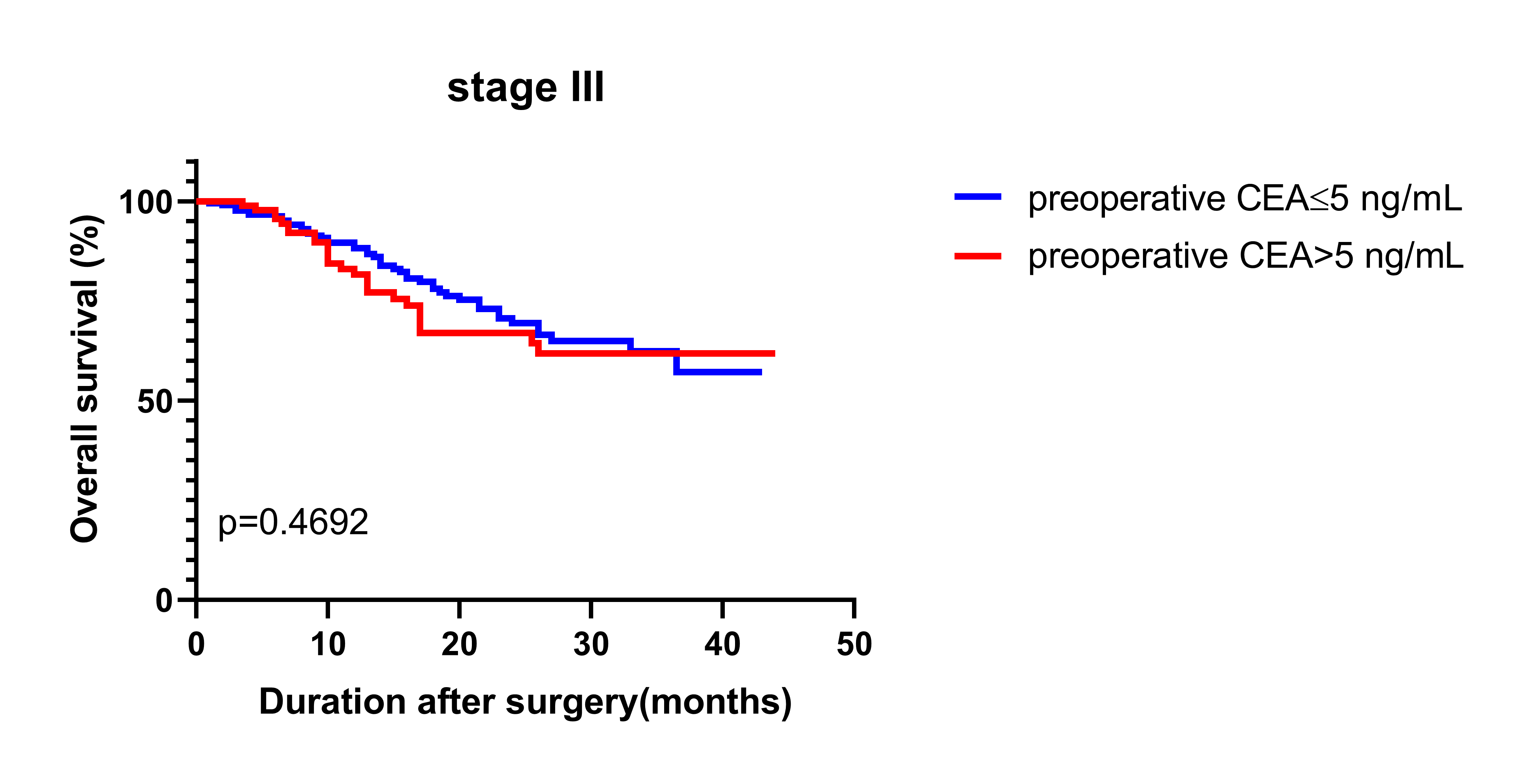


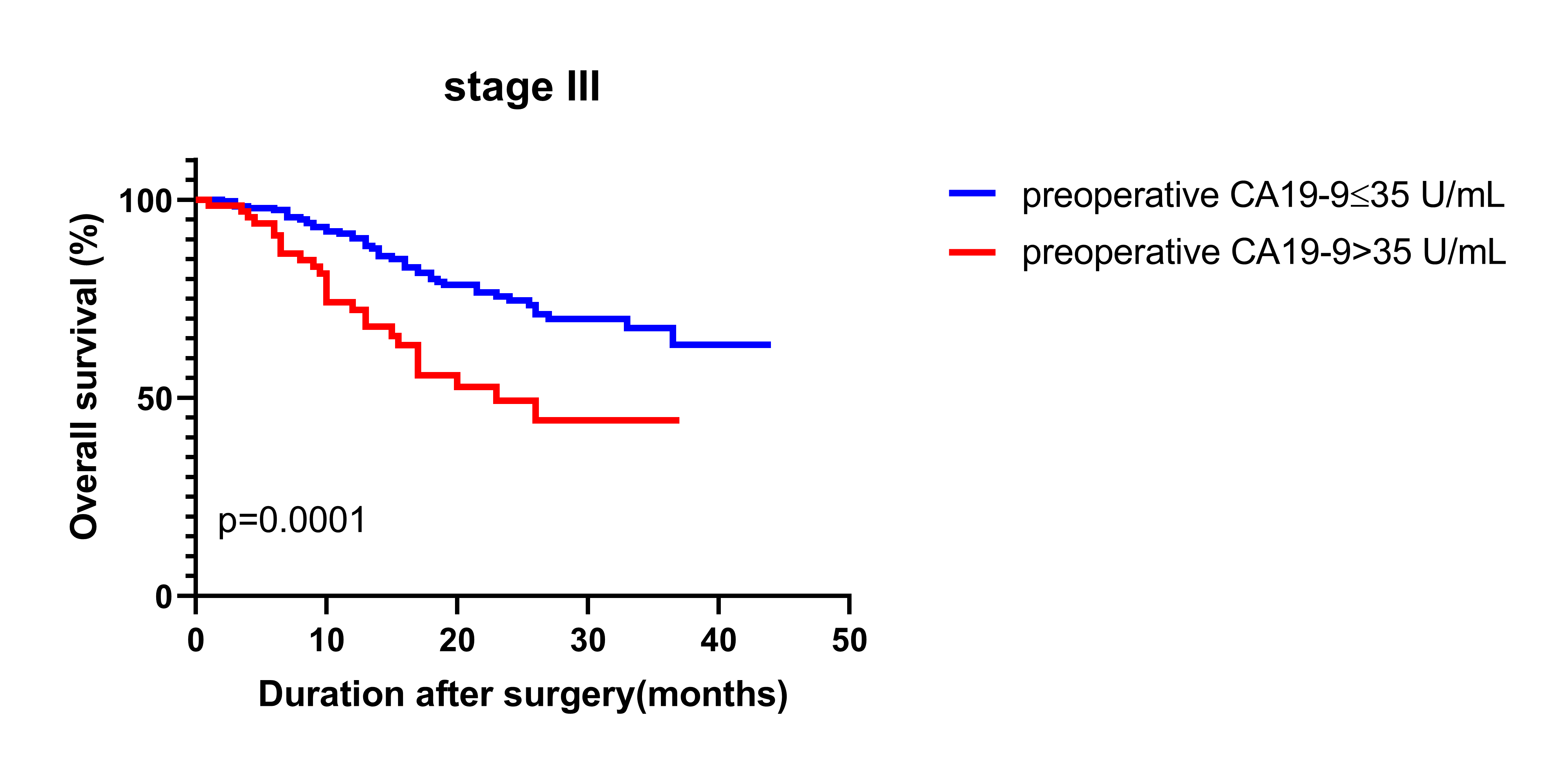


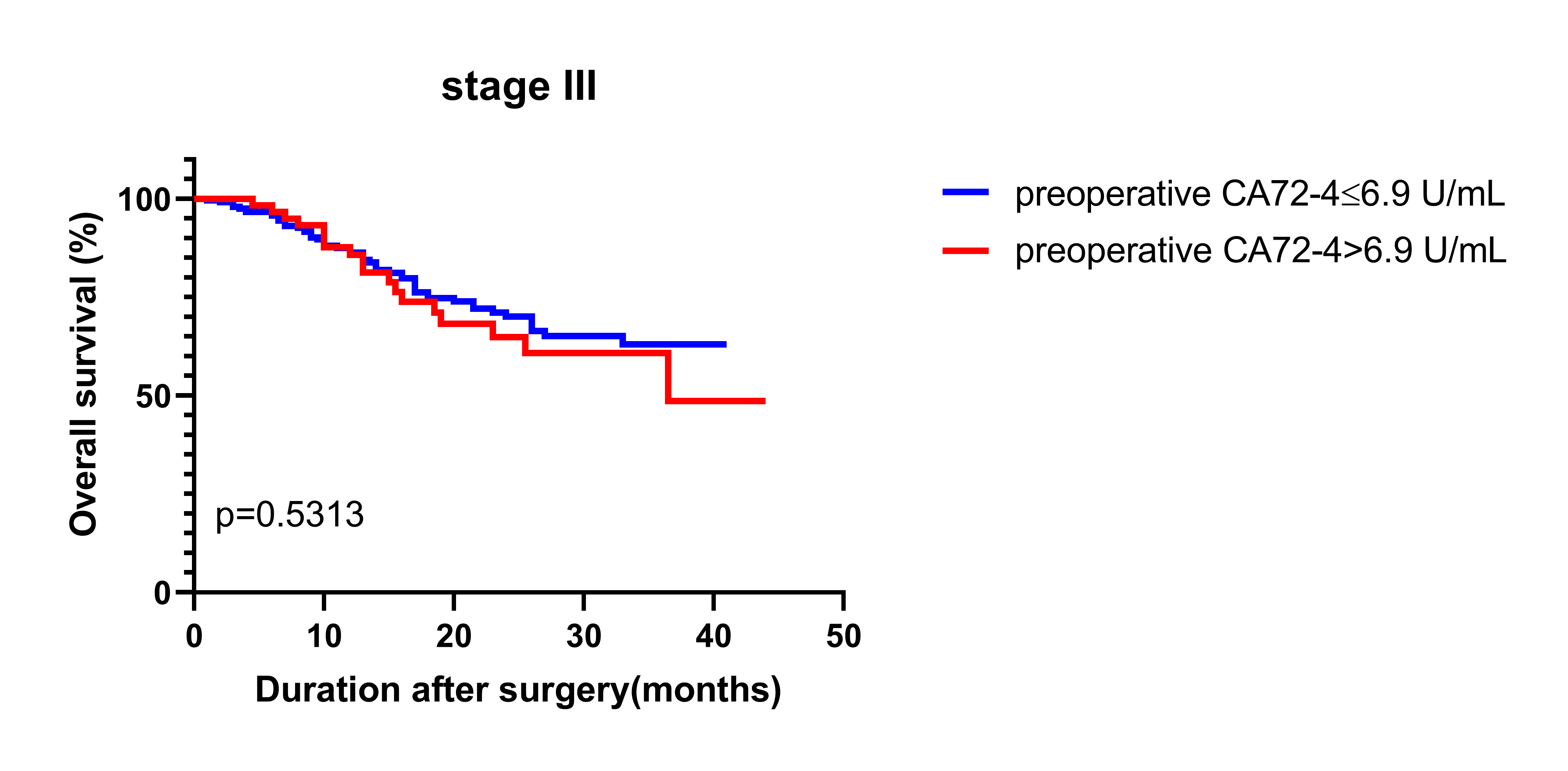


**B** preoperative level (RFS)


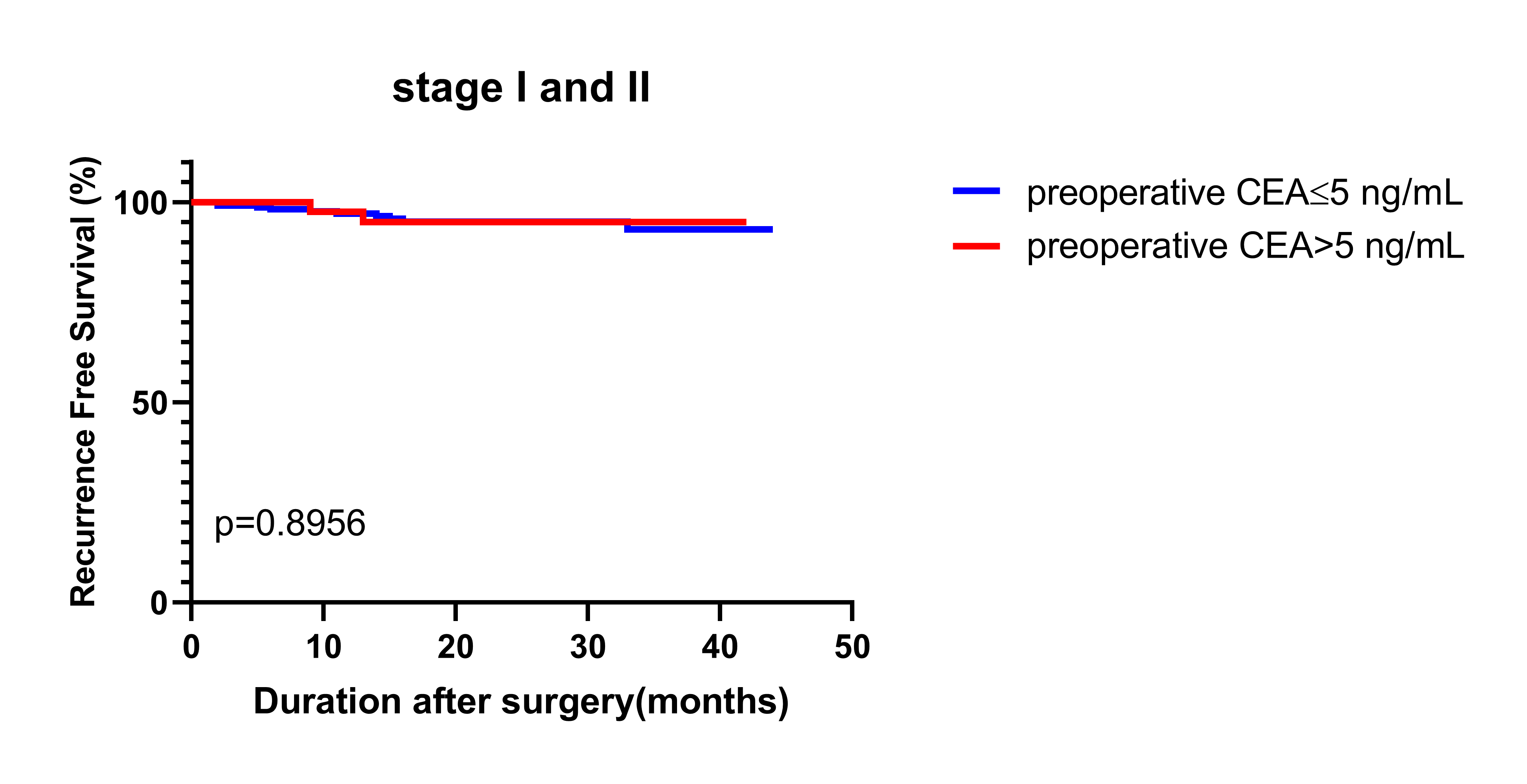


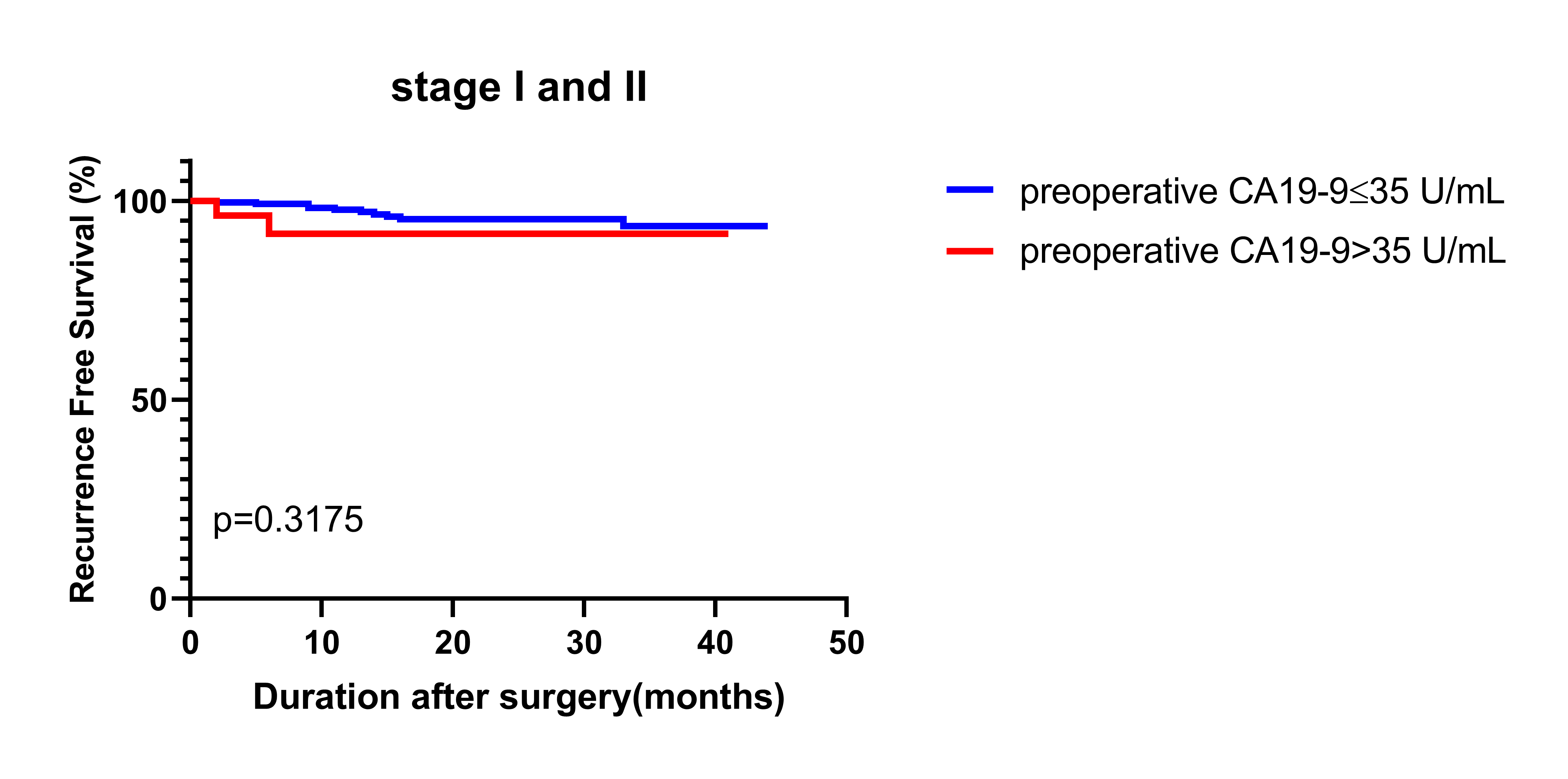


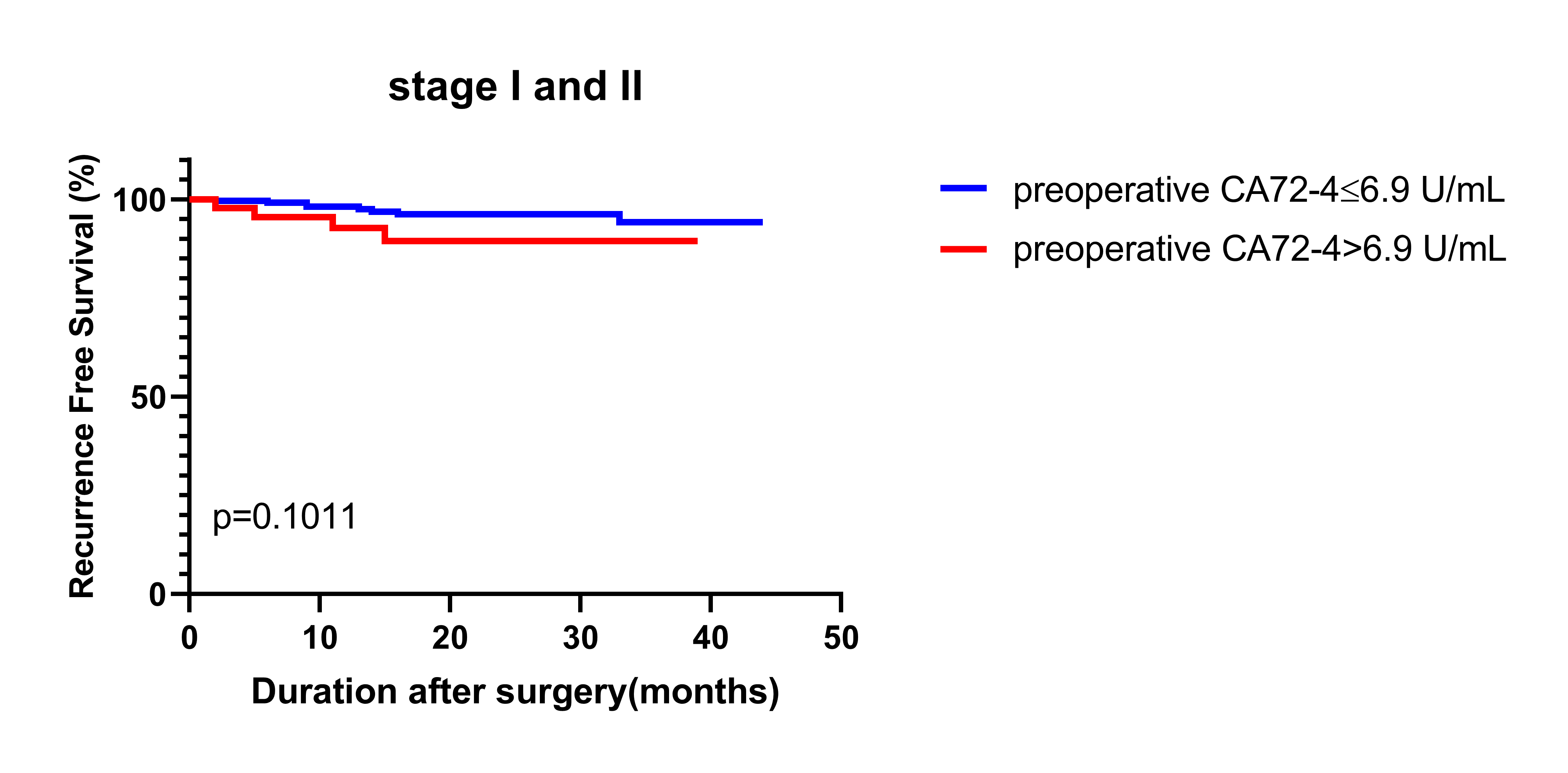


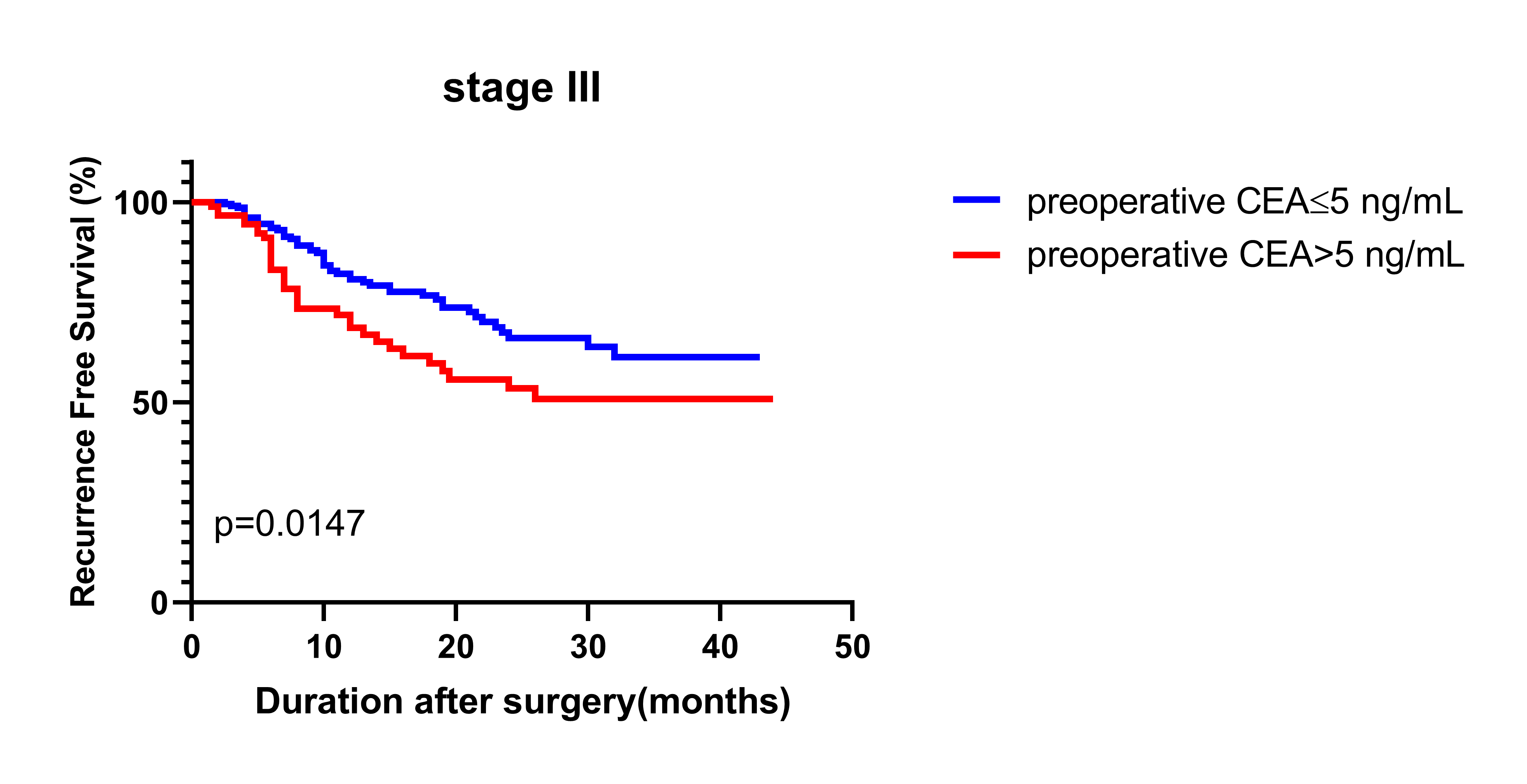


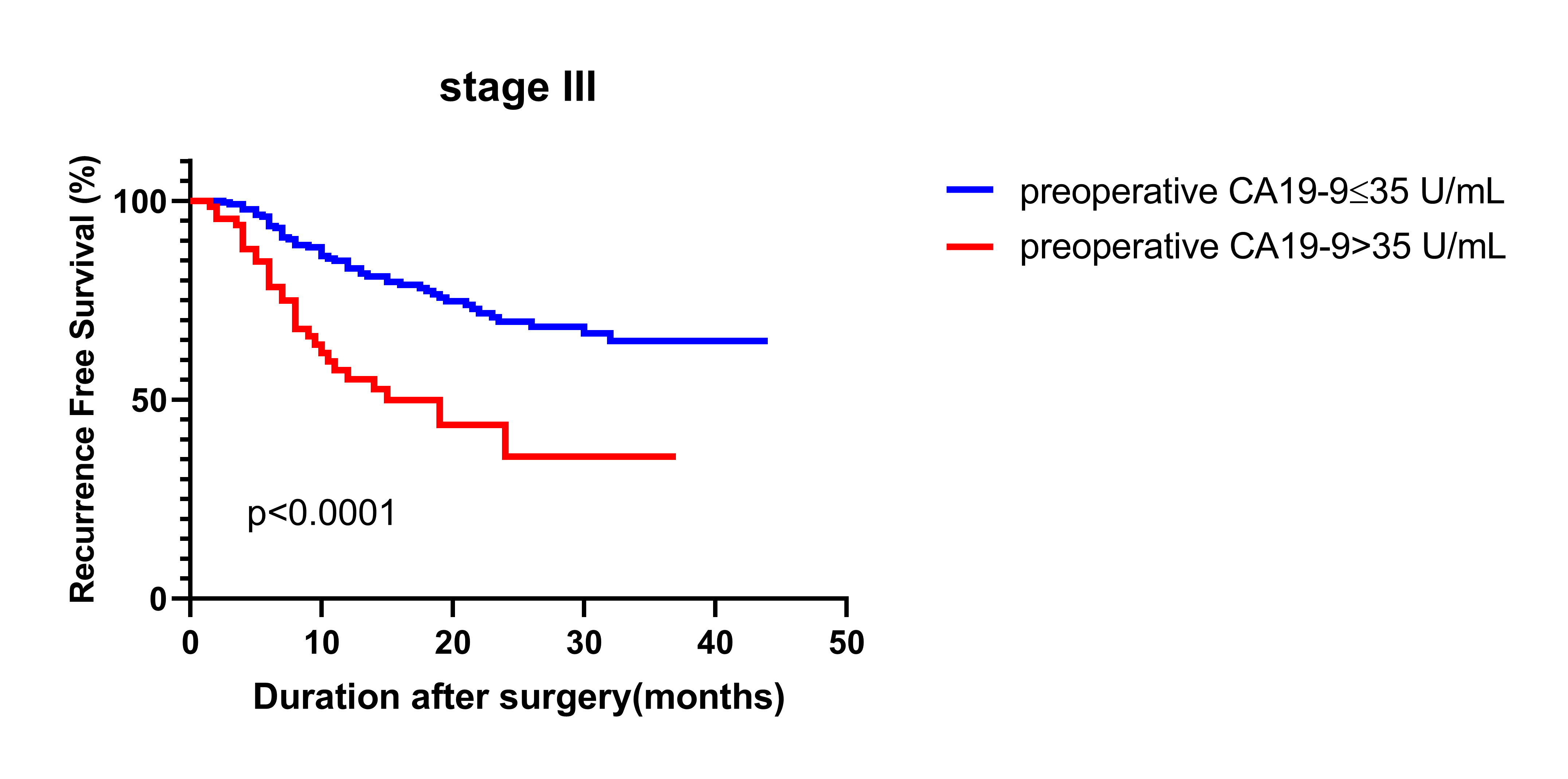


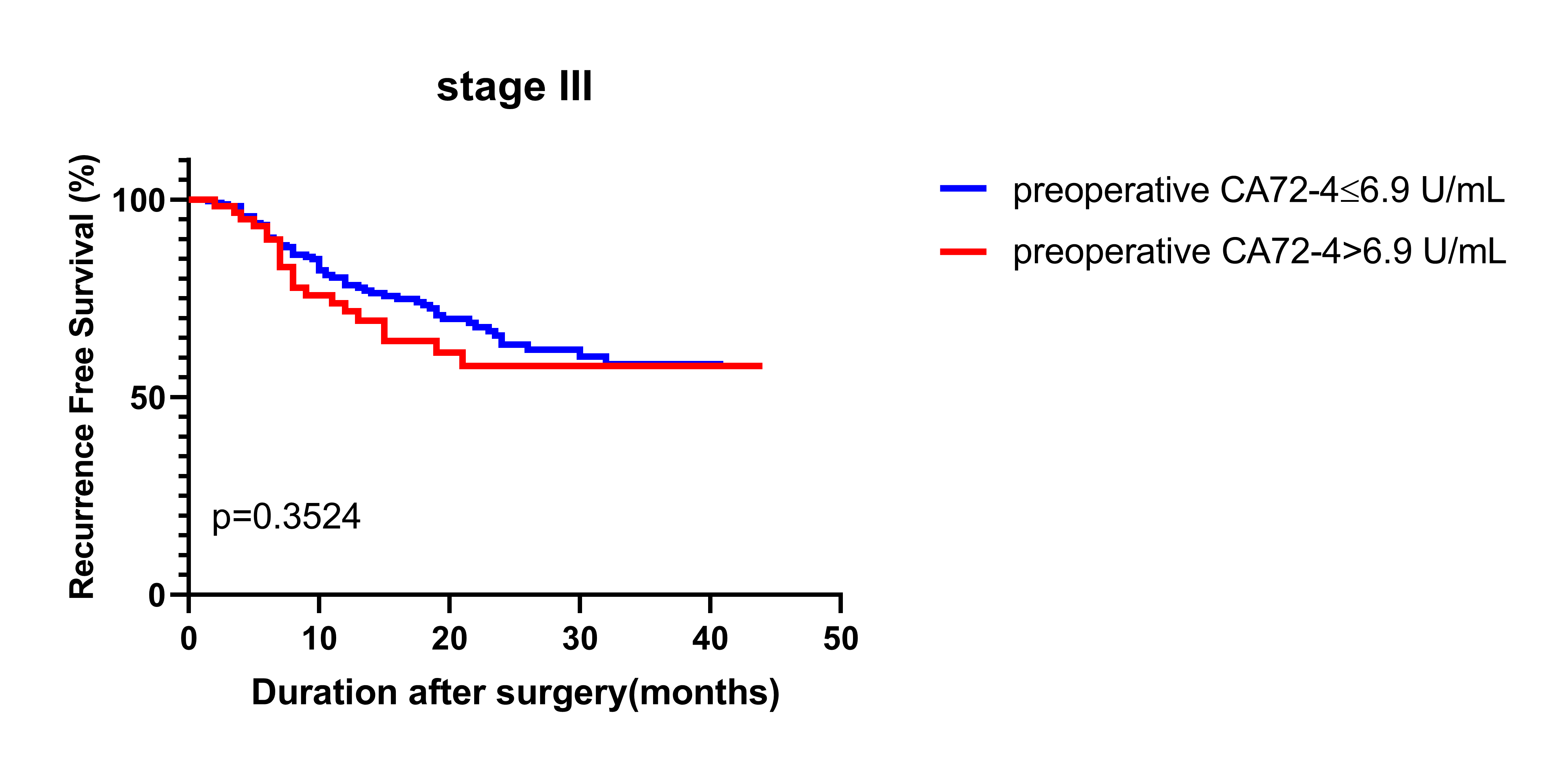


**C** postoperative level (OS)


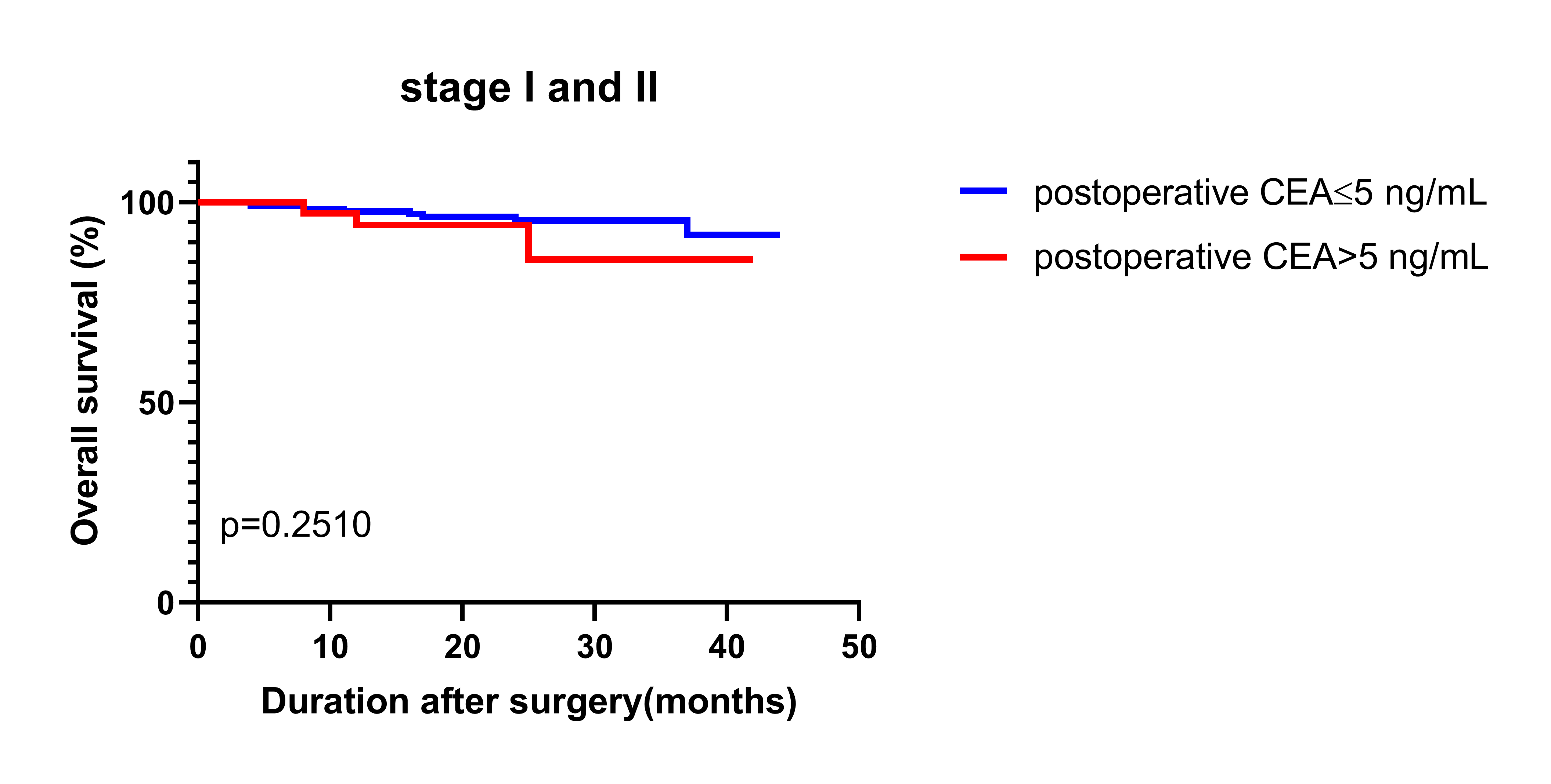


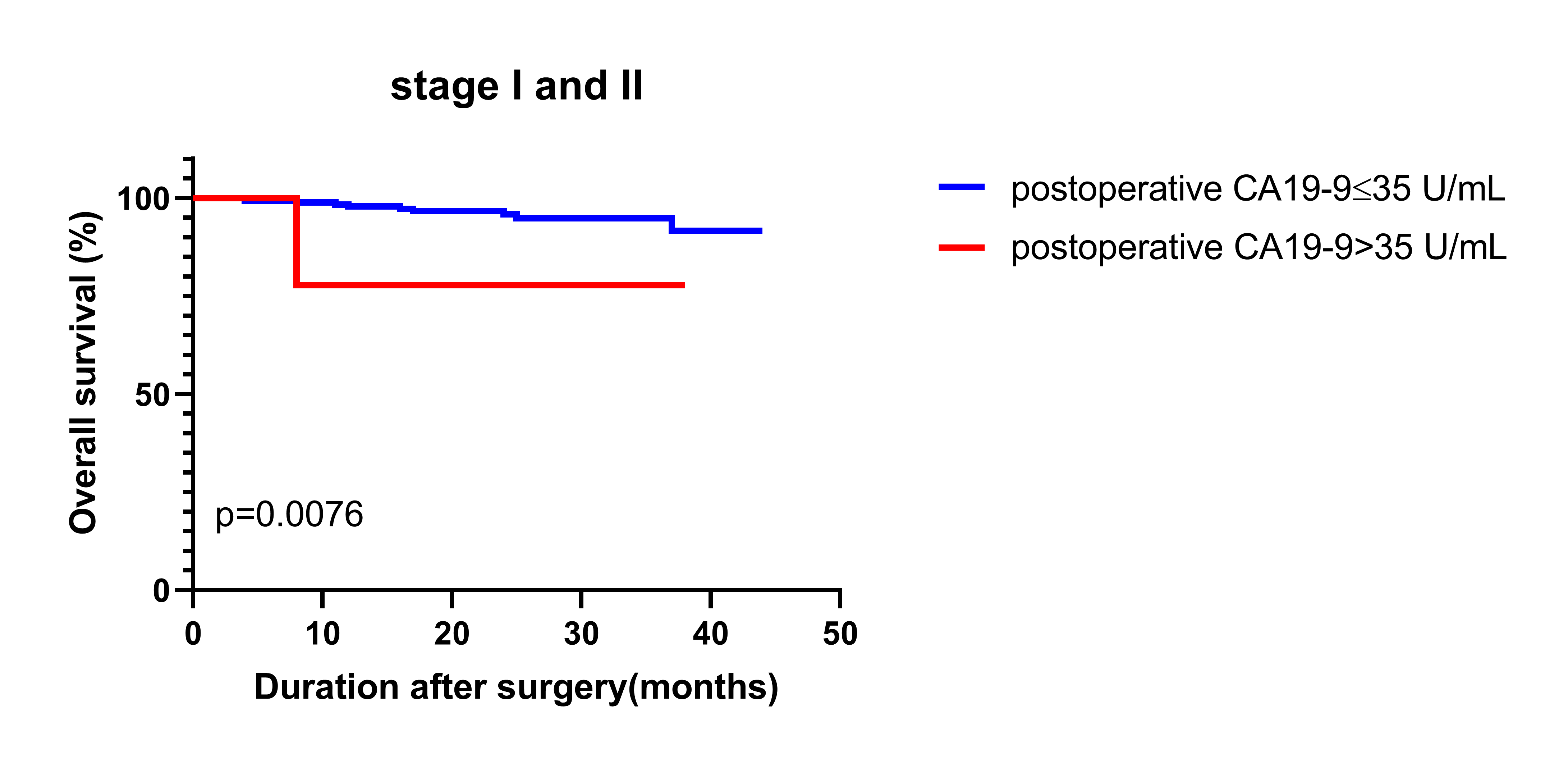


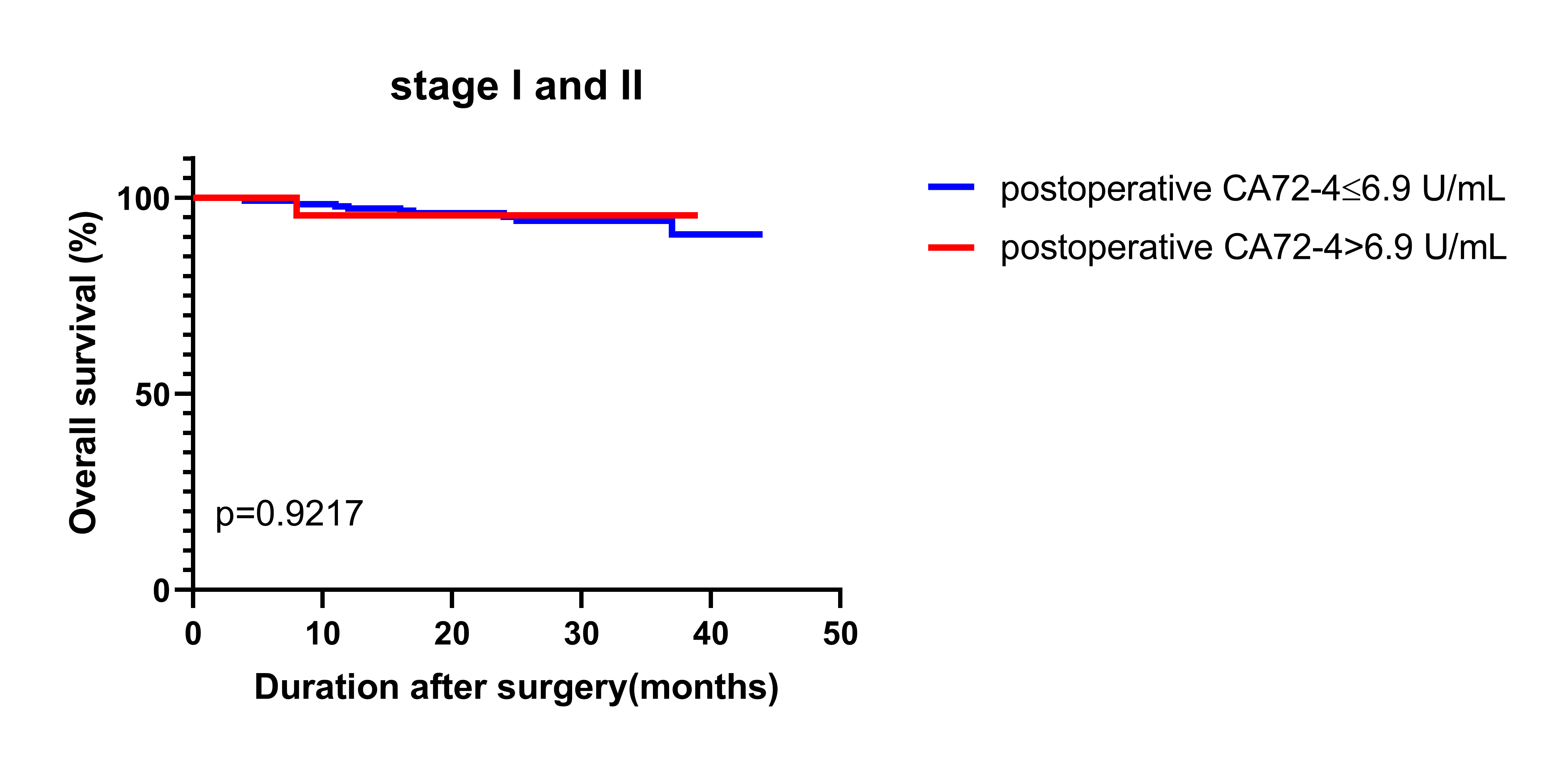


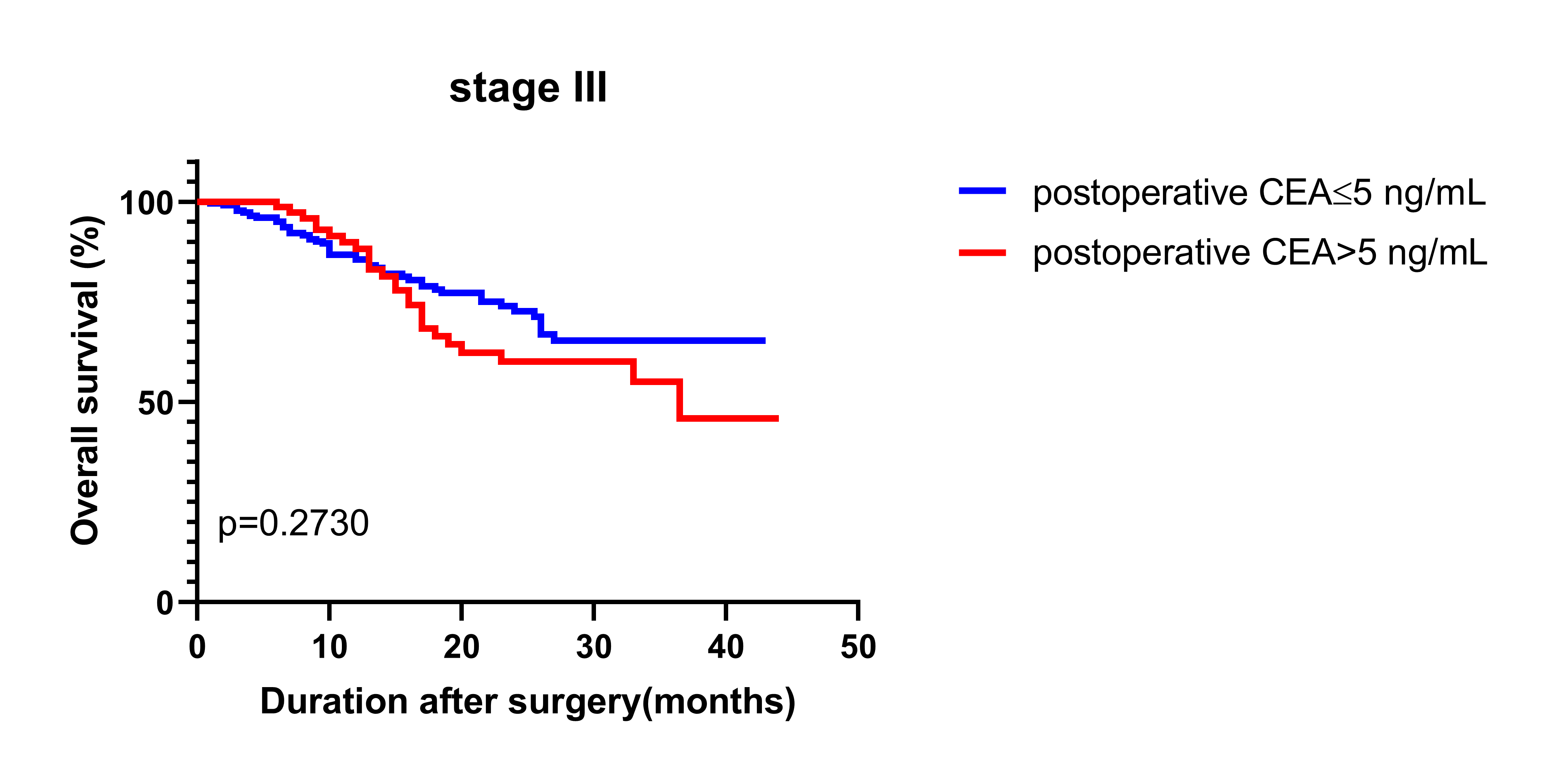


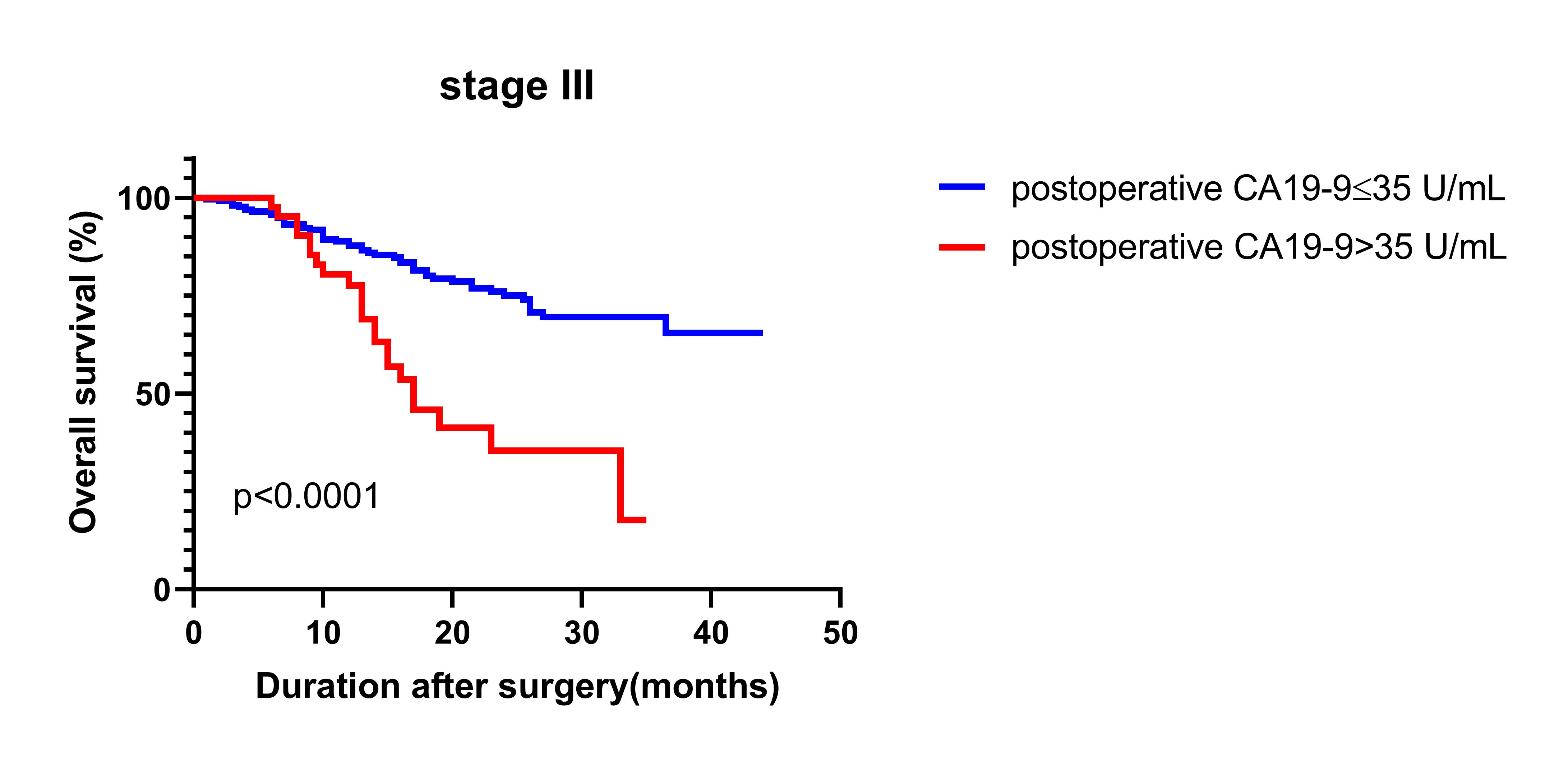


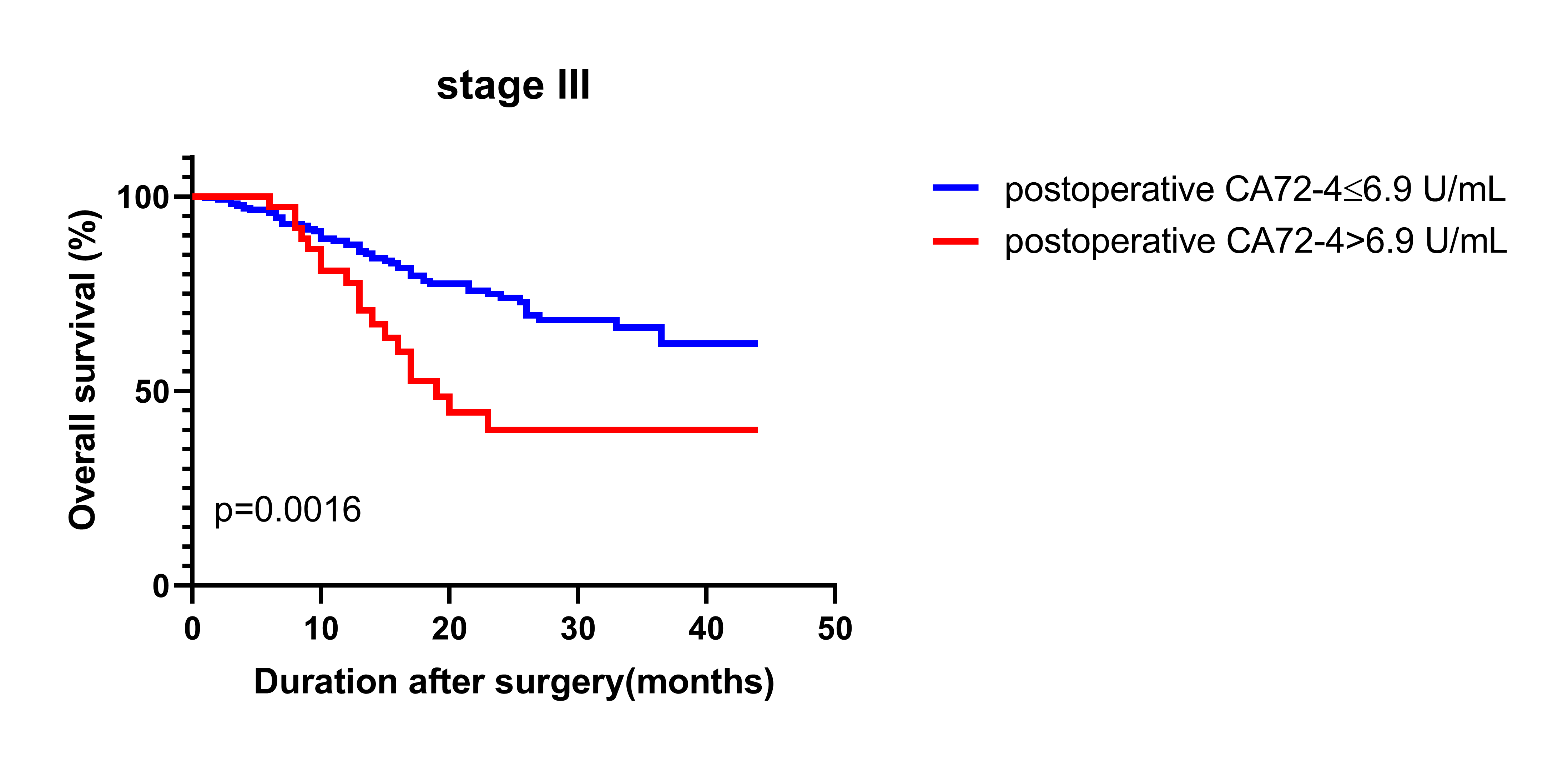


**D** postoperative level (RFS)


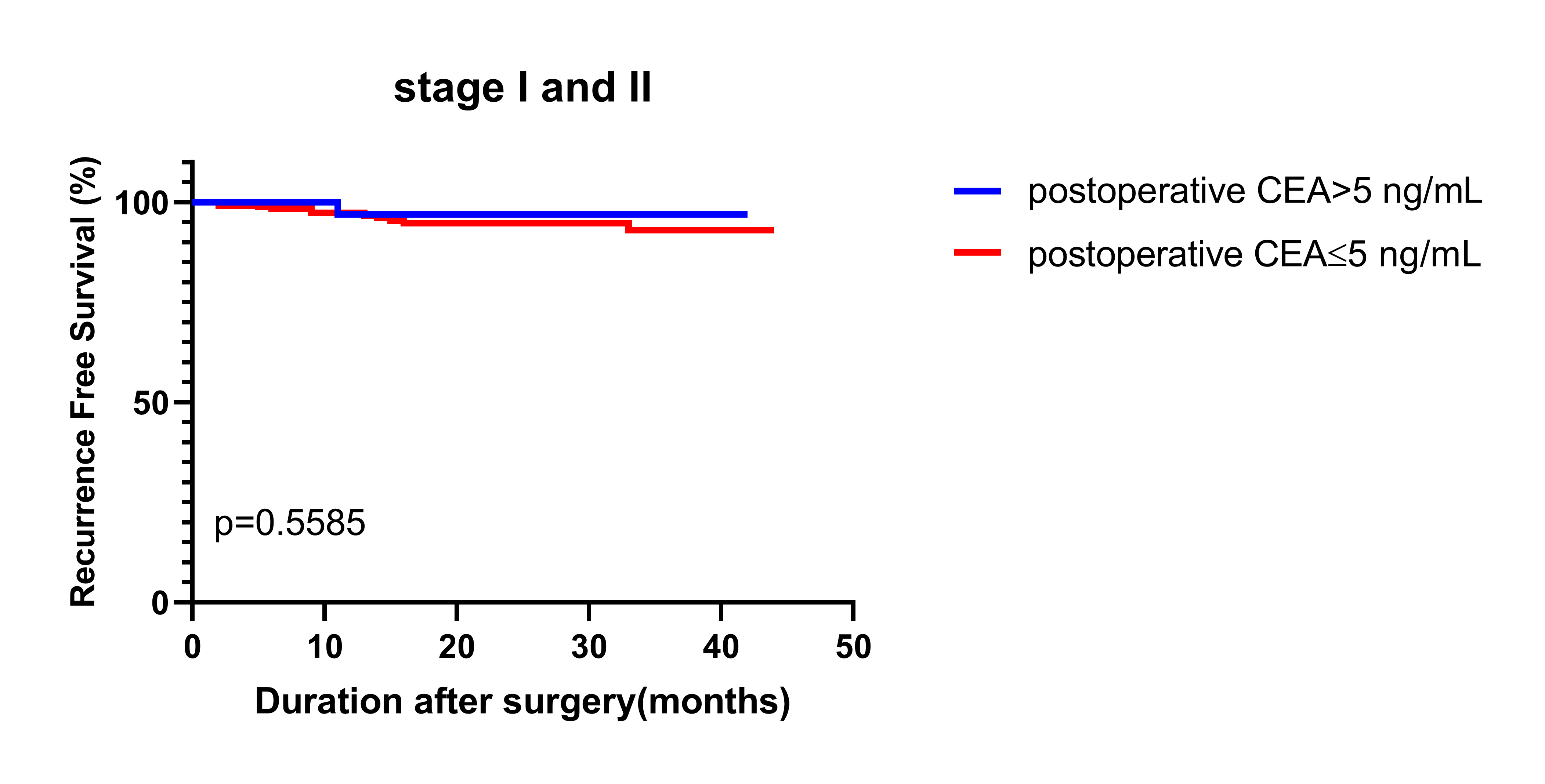


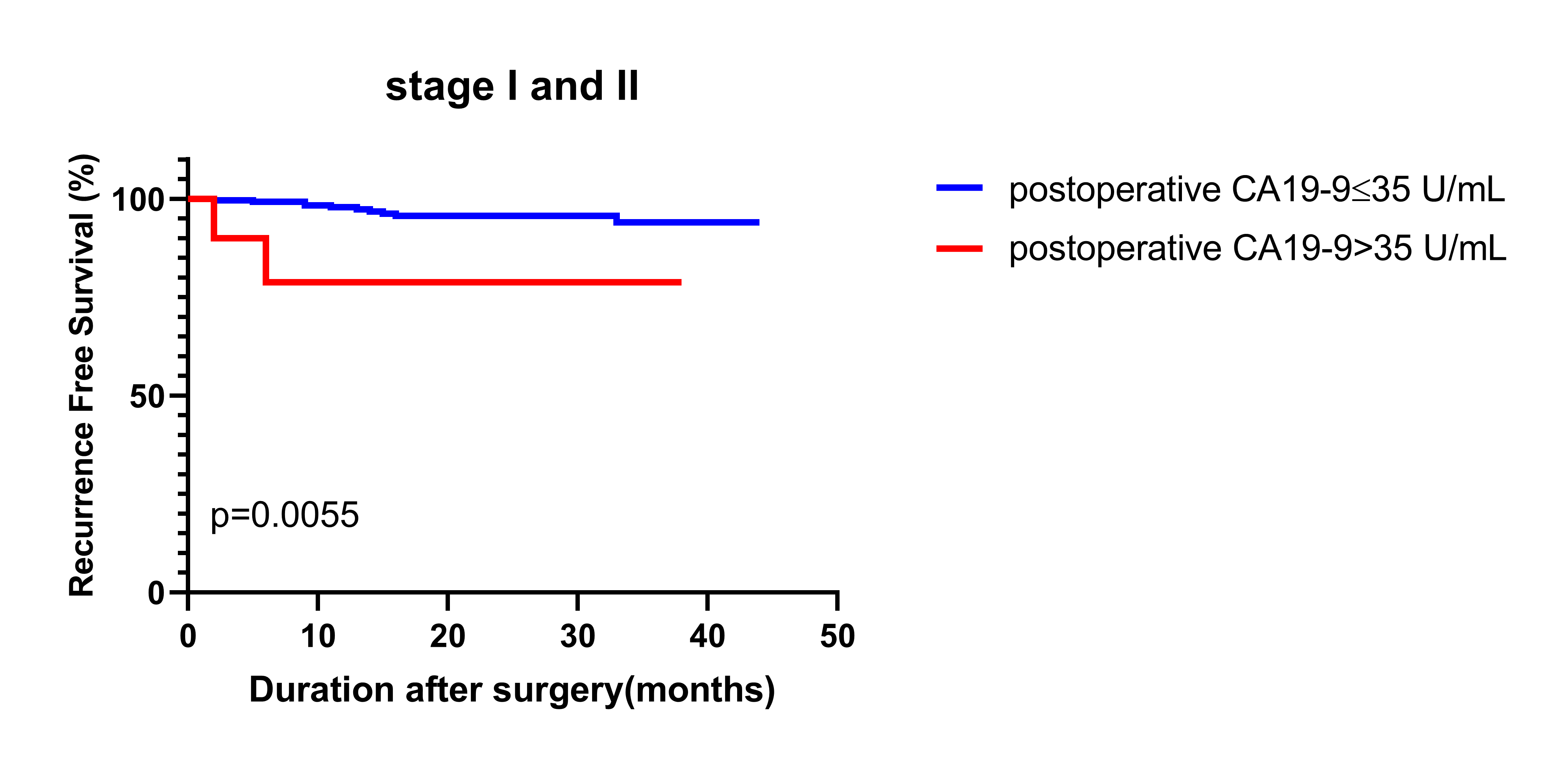


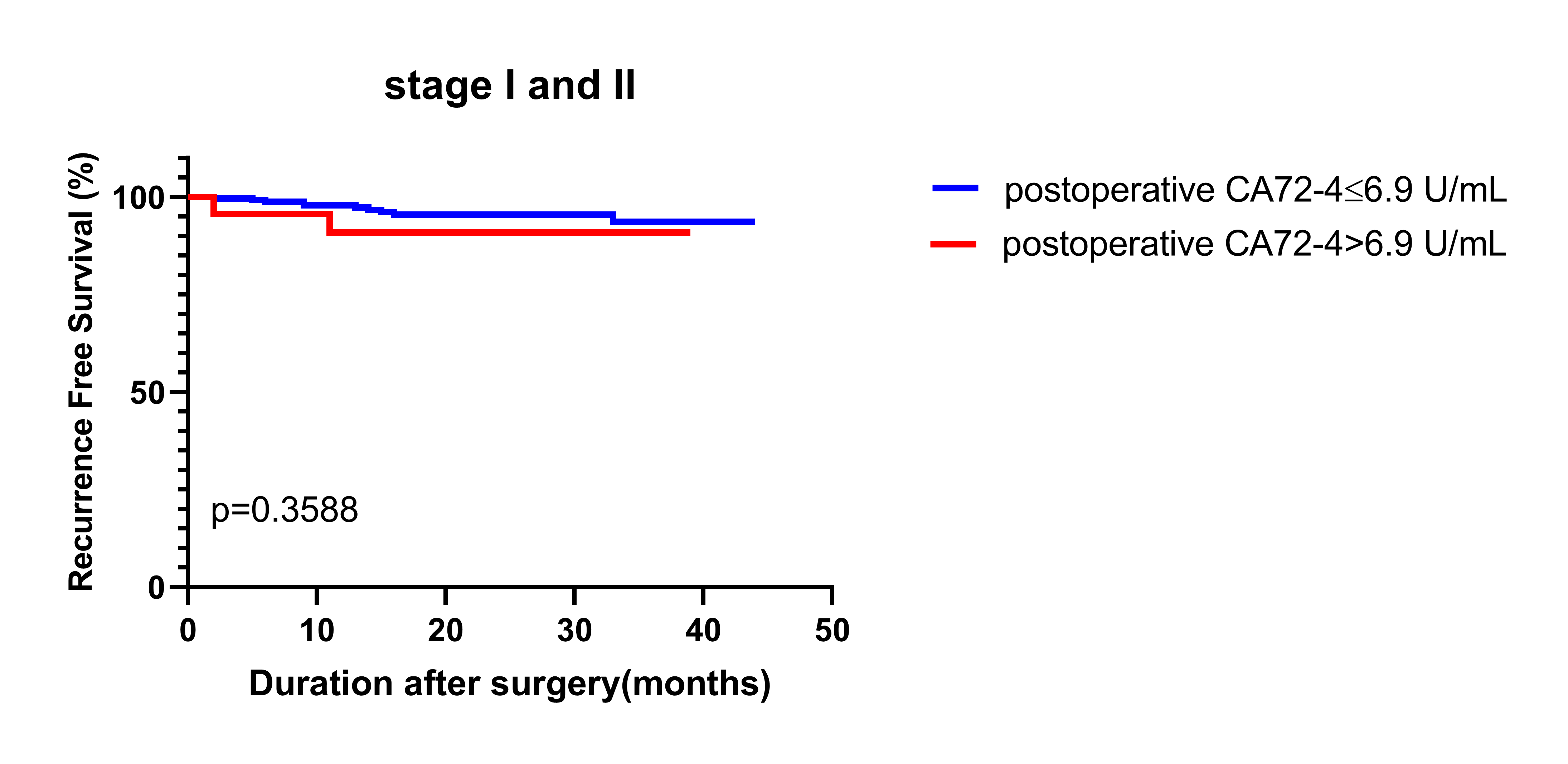


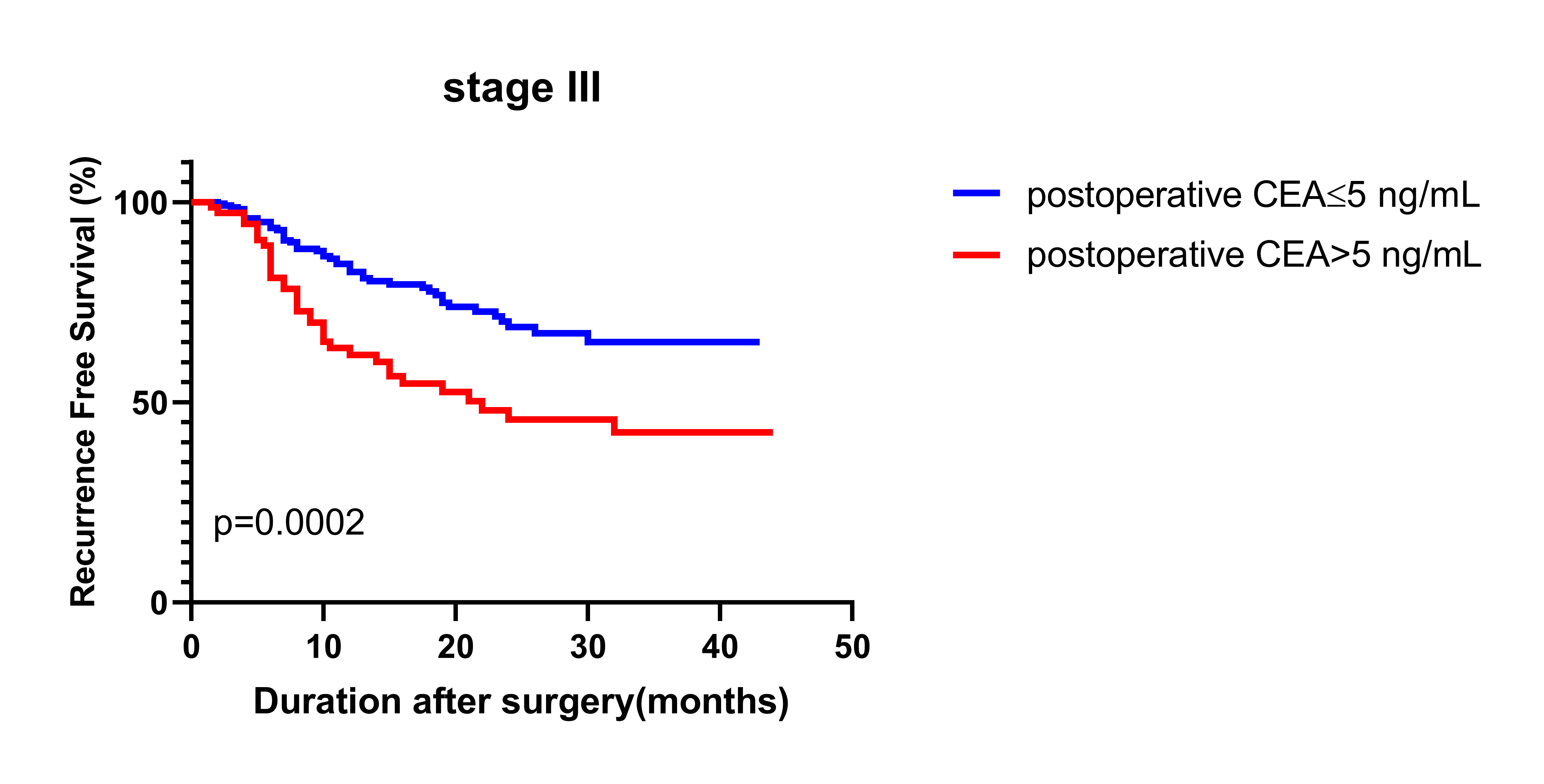


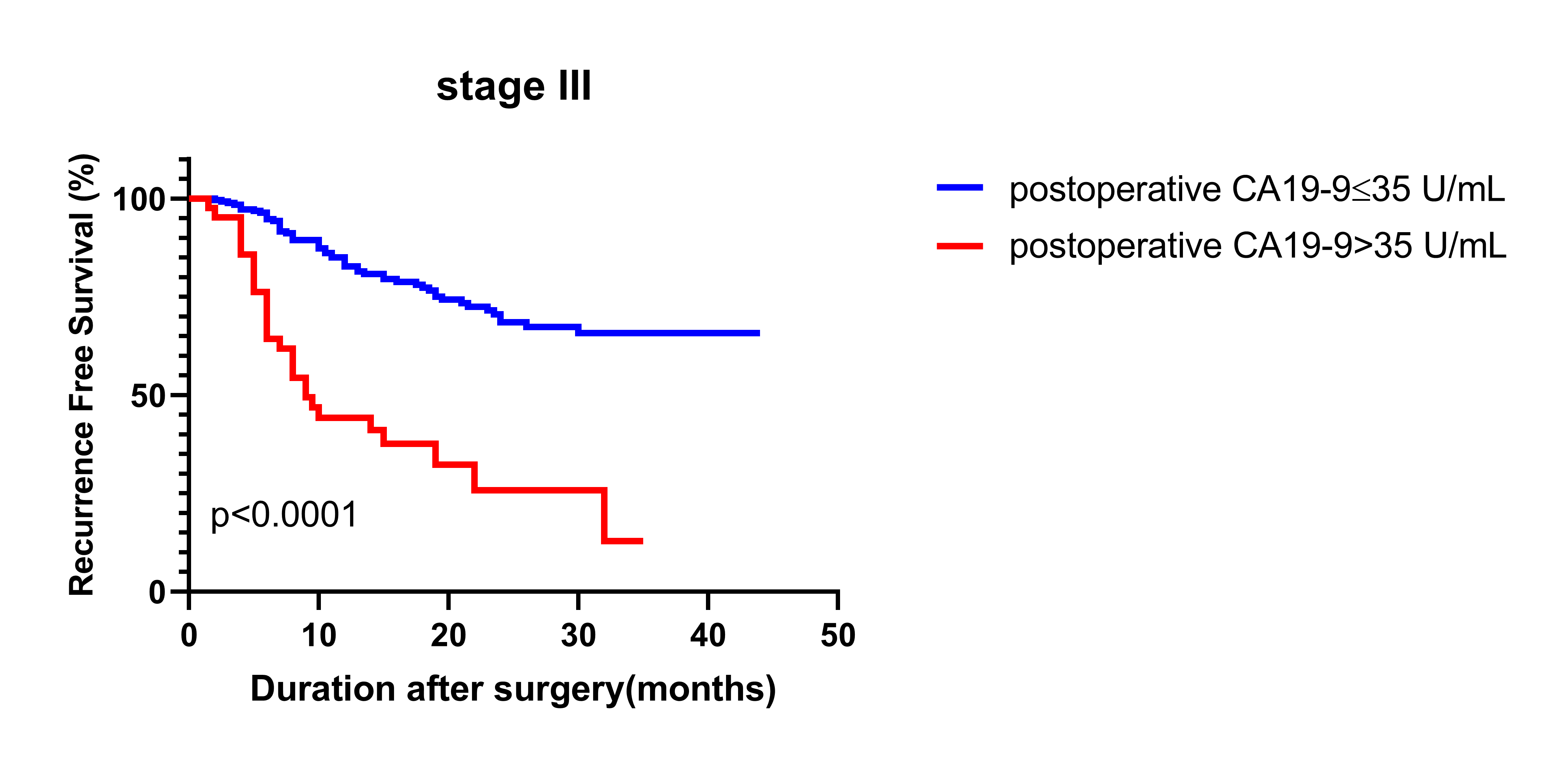


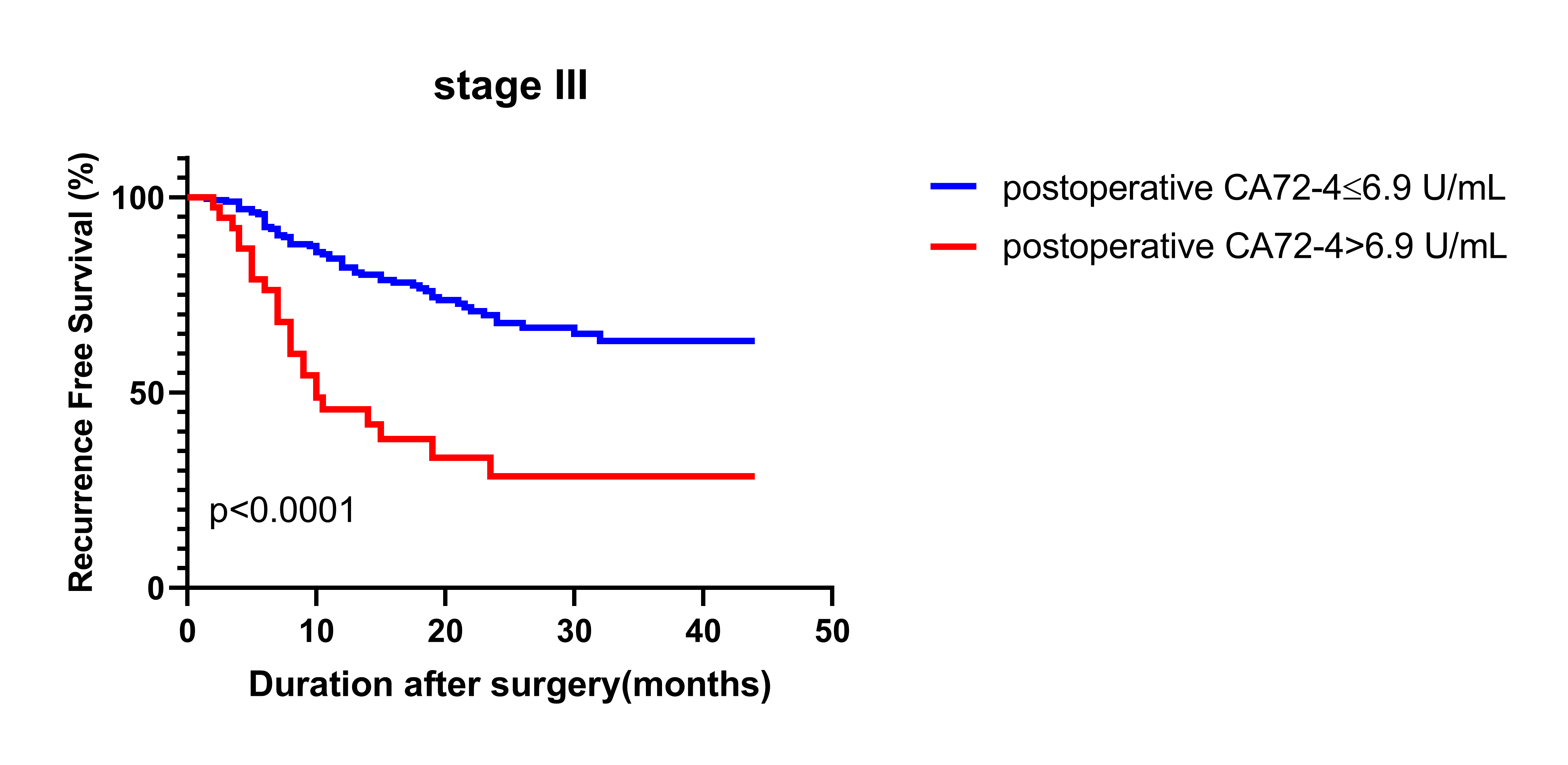


**Figure S** Prognostic impact of serum tumor markers. A and B: Overall and recurrence-free survival curves according to preoperative CEA, CA19-9 and CA72-4 levels; C and D: Overall and recurrence-free survival curves according to postoperative CEA, CA19-9 and CA72-4 levels. CEA: Carcinoembryonic antigen; CA19-9: Carbohydrate antigen 19-9. CA72-4:Carbohydrate antigen 72-4
